# Room temperature molecular up conversion in solution

**DOI:** 10.1038/ncomms11978

**Published:** 2016-06-15

**Authors:** Aline Nonat, Chi Fai Chan, Tao Liu, Carlos Platas-Iglesias, Zhenyu Liu, Wing-Tak Wong, Wai-Kwok Wong, Ka-Leung Wong, Loïc J. Charbonnière

**Affiliations:** 1 Laboratoire d'Ingénierie Moléculaire Appliquée à l'Analyse, IPHC, UMR 7178 CNRS, Université de Strasbourg, ECPM, Bât R1N0, 25 rue Becquerel, Strasbourg, 67087, France; 2 Department of Chemistry, Hong Kong Baptist University, Hong Kong SAR, Hong Kong; 3 Centro de Investigaciones Científicas Avanzadas, Departamento de Química Fundamental, Universidade da Coruña, Campus da Zapateira, Rúa da Fraga 10, A Coruña 15008, Spain; 4 State Key Laboratory of Chirosciences, Department of Applied Biology and Chemical Technology, Hong Kong Polytechnic University, Kowloon, Hong Kong; 5 Department of Applied Biology and Chemical Technology, Hong Kong Polytechnic University, Hong Kong SAR, Hong Kong

## Abstract

Up conversion is an Anti-Stokes luminescent process by which photons of low energy are piled up to generate light at a higher energy. Here we show that the addition of fluoride anions to a D_2_O solution of a macrocyclic erbium complex leads to the formation of a supramolecular [(ErL)_2_F]^+^ assembly in which fluoride is sandwiched between two complexes, held together by the synergistic interactions of the Er-F-Er bridging bond, four intercomplex hydrogen bonds and two aromatic stacking interactions. Room temperature excitation into the Er absorption bands at 980 nm of a solution of the complex in D_2_O results in the observation of up converted emission at 525, 550 and 650 nm attributed to Er centred transitions *via* a two-step excitation. The up conversion signal is dramatically increased upon formation of the [(ErL)_2_F]^+^ dimer in the presence of 0.5 equivalents of fluoride anions.

## Abstract (web summary)

Up conversion is an Anti-Stokes luminescent process by which photons of low energy are piled up to generate light at a higher energy. Here, the authors report a supramolecular assembly of fluoride-bridged erbium complexes which exhibit this behaviour in D_2_O solution at room temperature.

## Graphical abstract

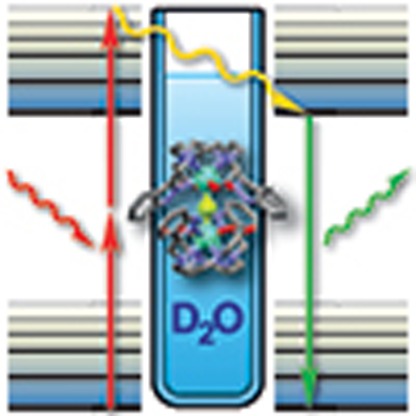

Conventional luminescence spectroscopy focuses on the emission of photons after absorption of photons of higher energy (that is, down shifting), but processes giving off emission of photons at higher energy than the incident beam and not related to thermal population of the excited states are far less common^1^. These processes gather aspects related to nonlinear optics, such as second and third harmonic generations or two photon absorption^2^, and those associated to the cumulative effects of multiple first-order absorption phenomena, also named up conversion (UC)^3^, among which are excited state absorption (ESA) and energy transfer up conversion (ETU)^1^. The last two became very popular in recent years, first because they are far more efficient than the others, affording observation of UC with more classical excitation sources, but also because of intensive research on the synthesis and preparation of UC materials, particularly in the field of lanthanide-doped phosphors^3^ and more recently with lanthanide-based nanoparticles^4^,^5^. Lanthanide ions such as Er, Tm or Ho are particularly well-suited for UC processes as they gather two important properties: a ladder-like energy level profile spanning from the Near-infrared (NIR) to the visible region; and long-lived excited states due to Laporte forbidden electric dipole transitions^6^.

UC is particularly appealing for biophotonic applications as the emitted signal is devoid of spurious signals such as auto-fluorescence of the sample or excitation bleed through, rendering an almost background-free measurement. Also, the NIR excitation is entirely dedicated to the excitation of the probe, as biological tissues absorb weakly in this region of the spectrum^7^, and the penetration of excitation light into the tissues is thus deeper, allowing applications to bioimaging^8^,^9^ or photodynamic therapy^10^.

Despite these evident new opportunities, the development of UC devices is still confronted with a scientific challenge: the downscaling of the probes to the molecular scale. In bulk solids and nanoparticles, the phonon energy of the lattice plays a crucial role in luminescence quenching mechanisms. When phonons or their overtones are in resonance with the intermediate excited state levels of the emitting centres, non-radiative phonon-assisted deactivation pathways compete severely with the successive piling up of excited states necessary to reach the higher energy emitting level^11^. At the molecular level, the situation is even worse, where OH, NH and CH oscillators, commonly present in the ligand backbones of the Ln complexes or in their solvation shells, contribute as efficient non-radiative deactivation pathways^12^. As a result, even conventional NIR luminescence of such Ln complexes rarely exceeds quantum efficiencies of a few percent, in the best cases^13^,^14^.

In spite of such apparently insurmountable obstacles, encouraging results were recently published. In 2011, Piguet, Hauser and co-workers reported on the observation of green erbium UC emission upon NIR chromium excitation in a heterotrinuclear triple-stranded helicate composed of two Cr sensitizers and a central Er acceptor in the solid state and solid solutions at room temperature and in frozen acetonitrile up to 150 K (refs 15, 16). Unambiguous evidence for the UC process was brought forward by the quadratic dependence of the emitted intensity as a function of excitation power. The study of isostructural compounds of the spectroscopically silent Ga analogue^16^ also allowed the authors to get a detailed overview of the kinetic parameters of their system. Within the frame of Förster's theory on energy transfer^17^, the energy transfer efficiency is dependent on the inversed sixth power of the donor–acceptor distance and one can anticipate that the rather large distances between the Cr sensitizers and the Er acceptor (8.9 Å on the basis of the analogous Yb compound) may not lead to an efficient energy transfer^18^. In 2012, Faulkner, Beeby and Sorensen achieved multiphoton excitation of different lanthanide salts of Eu, Tb and Sm upon intense excitation of some concentrated (*c*>0.01 M) triflate salts and complexes in d_6_-dimethylsulfoxide solutions^19^. In the case of thulium, they observed a faint excitation band in the multiphoton excitation spectrum that was proposed to be a potential UC process, but lacked details to follow up with the work^20^. Very recently, Hyppänen and co-workers reported on the observation of UC in a system composed of an Er complex and a NIR dye photosensitizer in CDCl_3_ at room temperature upon strong excitation (>3.4 W cm^−2^) at 808 nm (ref. 21).

In this contribution, we show that the macrocyclic complex of erbium with ligand L, [ErL(H_2_O)]^+^ can self-assemble in D_2_O solution in the presence of fluoride anions to form a supramolecular [(ErL)_2_F]^+^ dimer (Fig. 1). Upon excitation into the NIR absorption band of Er at 980 nm in D_2_O, a green emission is observed arising from an UC process, which is largely amplified with the formation of the Er dimer, representing, to the best of our knowledge, the first evidence of molecular UC in heavy water solution at room temperature.

## Results

### Synthesis and characterization of the complex

The [ErL(H_2_O)](NO_3_) complex was prepared from ligand L and Er(NO_3_)_3_.5H_2_O (ref. 22) and was fully characterized by elemental analysis, infrared spectroscopy, ^1^H-NMR and electrospray mass spectrometry (Supplementary Figs 1–4). The presence of the nitrate anions was particularly well evidenced by infrared spectroscopy with new bands at 1,655 and 1,360 cm^−1^, absent in the chloride complexes of Eu and Tb with L (ref. 22), and characteristic of nitrate anions^23^. In H_2_O, the electrospray mass spectrum of the complex displayed a single intense peak with maximum at 714.19 *m/z* units, with an isotopic distribution perfectly fitting that calculated for a [ErL]^+^ monocharged cation. A very minor species (<2%) could be evidenced as a peak with maximum at 1,488.37 *m/z*, which was related to the presence of two monomers linked by a nitrate anion [(ErL)_2_(NO_3_)]^+^ (calculated mass for C_56_Er_2_H_68_N_17_O_11_^+^: 1,488.39). Finally, the complex was also characterized by ^1^H-NMR spectrometry. Although at the limit of the solubility, and despite the paramagnetic contribution of the Er cation, it was possible to record the spectrum of the complex in D_2_O. The spectrum of the paramagnetic complex displayed 15 broad peaks between ca −110 and +110 parts per million (p.p.m.), for 16 expected for a *C*_*2*_ symmetry of the complex (Supplementary Figs 1 and 2). It is surmised that the missing peak is hidden under the peak of the partially deuterated water, HDO. Interestingly, minor peaks corresponding to a second paramagnetic species (<6%) could also be observed in D_2_O. Considering that the major species is in a square antiprismatic geometry, as observed for the parent Eu and Yb complex^22^, the minor species may be attributed to the presence of some twisted square antiprismatic conformer, as observed in the Ln complexes of 1,4,7,10-tetraazacyclododecane-1,4,7,12-tetraacetic acid (DOTA)^24^.

### Spectroscopic characterization of the complex

**Figure 2:**
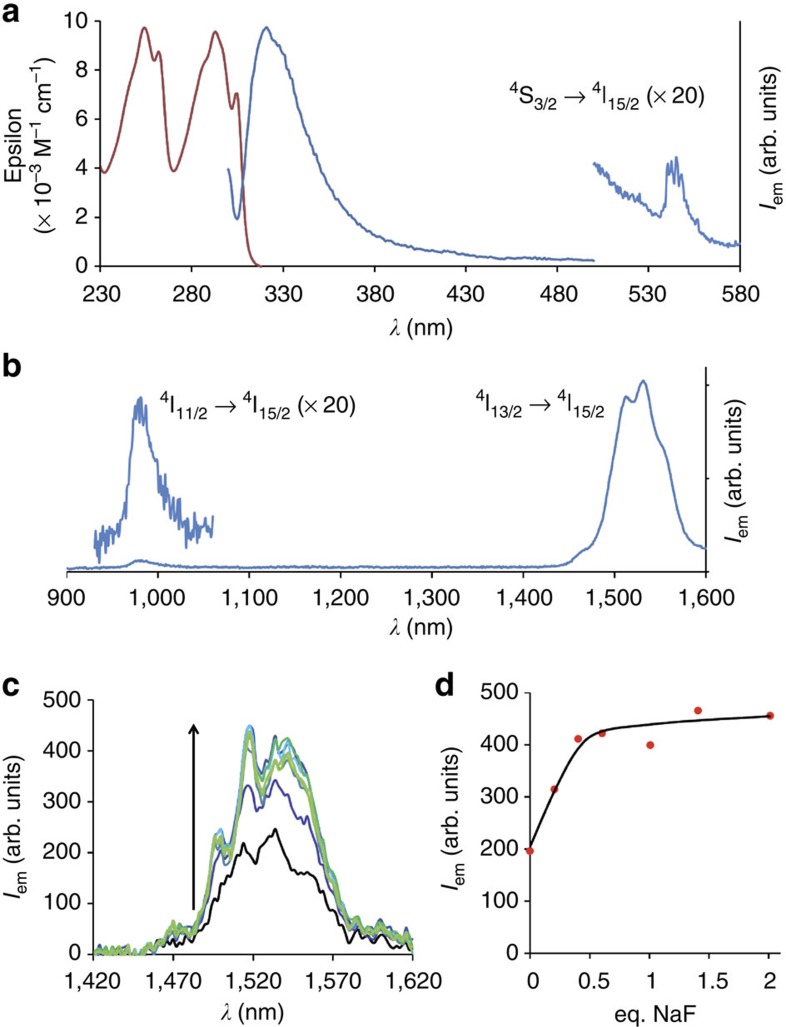
Spectroscopic properties of the Er complexes. (**a**) The ultraviolet–visible absorption spectrum of the Er complex in D_2_O (red, *c*=270 μM) displayed strong *π*→π* absorption bands. When excited into these bands (*λ*_exc_=294 nm), the emission spectrum of the complex displayed a ligand centred emission band in the visible region (blue), together with a contribution attributed to the ^4^S_3/2_→^4^I_15/2_ transition of Er (recorded at 0.5 nm resolution). (**b**) Other Er centred transitions can be observed in the NIR region (*λ*_exc_=294 nm, emission filter at 850 nm, D_2_O). (**c**) The addition of fluoride anions into the D_2_O solution of the Er complex resulted in an increase of the Er emission and (**d**) the intensity of the signal at 1,540 nm revealed a maximum increase for 0.5 equivalent (eq.) of fluoride anions, related to the formation of the dimeric [(ErL)_2_F]^+^ complex (the black curve corresponds to fitted data).

The absorption spectrum of the Er complex in D_2_O is composed of a broad absorption band with maximum at 294 nm (*ɛ*=9,850 M^−1^ cm^−1^, Fig. 2), which was attributed to *π*→π* transitions centred on the indazolyl moieties^25^. The transmittance spectrum was recorded in the solid state from 400 to 1,800 nm (Supplementary Fig. 5) and displayed numerous Er centred absorption bands attributed to transitions from the ground state to the ^4^F_7/2_, ^2^H_11/2_, ^4^S_3/2_, ^4^F_9/2_, ^4^I_11/2_ and ^4^I_13/2_, respectively at 488, 522, 541, 657, 980 and 1,514 nm.

Upon excitation into the ligand absorption bands (294 nm), the emission spectrum of the complex in D_2_O (Fig. 2) displayed a broad emission band, with maximum at 318 nm, which is attributed to some ligand centred fluorescence from the singlet state. A close examination of the visible part of the spectrum also revealed the presence of a structured emission peak between 540 and 550 nm in the low-energy tail of the fluorescence band, which can be ascribed to the ^4^S_3/2_→^4^I_15/2_ transition of Er. In the NIR region of the spectrum, a weak and broad band can be found at 980 nm, attributed to the ^4^I_11/2_→^4^I_15/2_ transition of Er and a strong and structured emission band could be observed with maximum at 1,535 nm associated to the ^4^I_13/2_→^4^I_15/2_ transition of Er. These last emissions arising from Er centred bands point to the presence of a partial ligand to metal energy transfer^26^. Performing the same measurement in H_2_O did not reveal any detectable Er emission in the NIR domain, nor in the 540–550 nm region. As previously observed for other lanthanides^22^, the replacement of the water molecule in the first coordination sphere of the complex by a D_2_O one has a dramatic influence on the luminescence properties, decreasing the non-radiative quenching of OH oscillators^27^.

Addition of fluoride to the D_2_O solution resulted in a *ca* 60% increase of the Er-centred luminescence intensity in the NIR region (Fig. 2c). At 1,540 nm, the maximum increase was observed at 0.5 equivalent of fluoride, corresponding to the formation of an Er_2_F species (Fig. 2d). As it was previously observed for measurements in D_2_O instead of H_2_O, the coordination of fluoride resulted in a supplementary decrease of the non-radiative quenching processes with a concomitant increase of the luminescence. The addition of larger amounts of fluoride resulted in minor changes in the luminescence intensity, pointing to very similar metal-centred luminescence quantum yields for the species present in solution.

### UC in D_2_O solution

**Figure 3:**
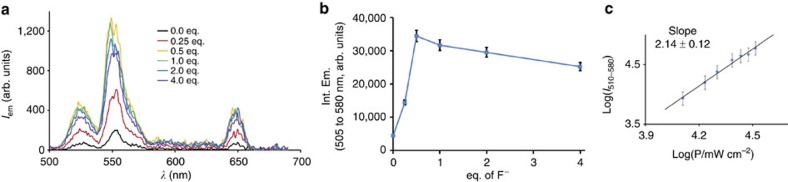
UC emission spectroscopy. Excitation of a 1 mM solution of the Er complex in D_2_O into the Er ^4^I_15/2_→^4^I_11/2_ absorption band at 980 nm revealed (**a**) a weak UC emission signal in the visible region associated to Er emission (black curve). Upon titration by fluoride anions, the UC emission strongly increased up to 0.5 equivalent (eq.) of fluoride (orange), as revealed (**b**) by the evolution of the intensity increase (integrated from 505 to 580 nm) as a function of added fluoride anions. (**c**) Plotting the UC emitted intensity (integrated from 505 to 580 nm) as a function of the incident pump intensity at 980 nm in a Log-Log format for the solution containing 0.5 eq. of fluoride anions gave a slope which corresponds to a two photon process (Errors were estimated assuming a Poisson's law for the intensities).

The possibility of an UC process with the Er complex in D_2_O was investigated. A 1 mM solution of the complex in D_2_O was irradiated at 980 nm into the Er ^4^I_15/2_→^4^I_11/2_ transition and the emission spectrum of the solution was recorded from 500 to 680 nm (Fig. 3). In the absence of fluoride, the UC spectrum displayed three weak emission bands at *ca* 525, 550 and 650 nm (Fig. 3a). These transitions were, respectively, assigned to the ^2^H_11/2_→ ^4^I_15/2_, ^4^S_3/2_→^4^I_15/2_ and ^4^F_9/2_→^4^I_15/2_ emission band from Er. To this solution, increasing amounts of fluoride anions were added in the form of NaF salt. After each addition, the mixture was agitated for 30 min to ensure the equilibration of the solution. As soon as fluoride was added, the intensity of the peaks strongly increased, with a maximum at 0.5 equivalent of added fluoride, and slowly decreased afterwards (up to 4 equivalents). Interestingly, the intensity of the peaks was clearly related to the formation of an Er_2_F species, with a 7.7-fold increase at 0.5 equivalent compared with the fluoride-free solution (Fig. 3b).

To further characterize the UC process, the luminescence intensity of the ^2^H_11/2_→ ^4^I_15/2_ and ^4^S_3/2_→^4^I_15/2_ transitions from 505 to 580 nm was monitored as a function of the intensity of the incident beam at 980 nm (Fig. 3c) for a mixture containing 0.5 equivalent of fluoride anions (Supplementary Fig. 6)^22^. The calculated slope (2.14±0.12, obtained as the average value of four independent measurements) is slightly larger than the expected value of 2, in good agreement with UC processes^28^, but such a slope may also be related with a possible ligand-based nonlinear process followed by sensitization of Er. To exclude this last possibility, the spectrum recorded at 0.5 equivalent of added fluoride was enlarged down to the ultraviolet region (from 350 to 700 nm, Supplementary Fig. 7). In these conditions, only the above-mentioned Er centred emission bands could be observed with no traces of ligand centred emission, as would be expected for a ligand-based nonlinear process followed by Er emission, similar to what is observed with one photon excitation of the ligand at 294 nm (Fig. 2). Also, the possibility of an emission at 545 nm resulting from a spurious signal arising from the ^5^D_4_→^7^F_5_ transition of Tb impurities can be excluded by comparing the measured UC spectrum with that of the analogous Tb complex (Supplementary Fig. 8). Finally, the intensity of the UC signal at 545 nm was monitored by varying the excitation wavelength from 700 to 1,050 nm (Supplementary Fig. 9), showing a maximum of UC excitation at 980 nm for the ^4^I_15/2_→^4^I_11/2_ transition, thereby excluding the possibility of a nonlinear multiphoton process. Considering the relative weakness of the observed signal, an estimation of its quantum efficiency could be made by comparison with Er-doped UC nanoparticles pointing to an upper limit of 10^−5^.

Additional titration experiments were performed to monitor the one photon emission spectra during Er titration with NaF with excitation into the ligand (294 nm). The visible part of the spectrum showed a weak increase of the ligand centred emission upon fluoride addition, but poor to negligible changes of intensity of the ^4^S_3/2_→^4^I_15/2_ transition of Er (at *ca* 550 nm, Supplementary Fig. 10). Following the NIR emission of the ^4^I_13/2_→^4^I_15/2_ transition of Er (1,450 to 1,600 nm, Supplementary Fig. 11) upon 980 nm excitation (^4^I_15/2_→^4^I_11/2_) resulted in a maximum increase of ca 70% of the emission intensity, similar to what is obtained upon ligand centred excitation (Supplementary Fig. 12). Altogether, these results showed that the intensity increase of the one photon emission is not appreciably related to an improved ligand to metal energy transfer efficiency, but clearly to a better protection of the Er cation towards non-radiative processes. The weak intensity increase of the ^4^S_3/2_→^4^I_15/2_ transition compared with that of ^4^I_13/2_→^4^I_15/2_ may be related to important changes in the crystal field splitting of Er (ref. 29) with concomitant changes in the branching ratio.

### Speciation of the species formed in solution

The formation of the new species was first monitored qualitatively by electrospray mass spectrometry analysis of the mixtures. Addition of 0.5 equivalent of sodium fluoride to the complex (Supplementary Figs 13 and 14) led to a new peak at 1,449.46 *m/z* units, which was attributed to a [(ErL)_2_F]^+^ singly charged dimer, together with a doubly charged cation with a peak maximum at 736.23 *m/z* units, attributed to the sodium adduct [(ErL)_2_F+Na]^2+^. It is interesting to note that each complex is always present with two deuterium atoms, likely as a result of the H–D exchange of the imidazol protons in D_2_O. At 1.0 equivalent of NaF (Supplementary Fig. 15), the peak of the singly charged dimer at 1,449.46 became intense in the spectrum, whereas the peak corresponding to the [ErL]^+^ monomer almost vanished. The other important species were also attributed to the dimer and can be observed with maxima at *m/z* of 725.23 and 736.22, corresponding to the doubly charged dimer with a proton or a sodium cation, respectively. At two equivalents of fluoride (Supplementary Fig. 16), peaks corresponding to the singly charged and doubly charged dimer at 1,449.45 and 736.22 *m/z* units are still present, and the peak at 716.23, assigned to the [ErL]^+^ complex became important. In these conditions, it is expected that the neutral [ErLF] monomer began to form in solution, and its ionization resulted in the breaking of the Er–F bond, releasing the [ErL]^+^ species.

**Figure 4:**
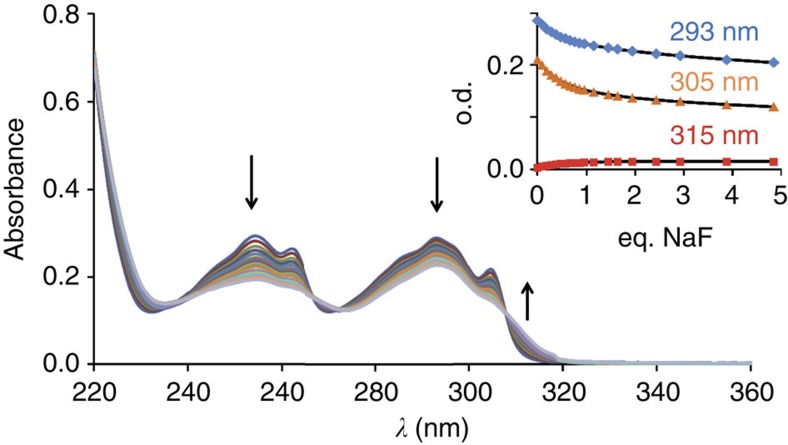
Ultraviolet–visible titration of [ErL(H_2_O)](NO_3_). The progress of the dimerization of the erbium complex was followed by ultraviolet–visible absorption spectroscopy by addition of NaF (0–4.8 eq.) to a solution of [ErL(H_2_O)](NO_3_) (*c*=3.15 × 10^−5^ M) in H_2_O. The evolution of the spectra can be fitted with equations (1) and (2) (see the text) and the comparisons between measured and calculated values (black lines) of the absorbances at 293, 305 and 315 nm are presented in the inset.

The formation of the [(ErL)_2_F]^+^ complex was also monitored by ultraviolet–visible absorption spectroscopy, following the evolution of the absorption spectra of the complex in the presence of increasing amounts of fluoride (Fig. 4) in H_2_O solution.

The association of fluoride was fitted to the equilibria:


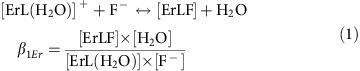



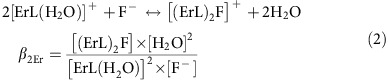


Taking the water concentration as constant during the titration, analysis of the ultraviolet–visible absorption data by the Specfit software^30^ using equations (1) and (2) afforded stability constants of 6.1(3) and 13.0(2) log units, respectively, for *β*_1Er_ and *β*_2Er_. The results for the cumulative stability constant *β*_2Er_ are in excellent agreement with the one previously reported for other complexes (log *β*_2Ln_=13.0(3), 12.5(1.0) and 12.6(1.0), respectively, for Eu, Tb and Yb)^22^. In contrast to what was observed for the other lanthanide cations, the titration of the Er complex allows the observation of the mononuclear [ErLF] intermediate complex. It is worth noting that the ratio of the stepwise association constants, *K*_2Er_/*K*_1Er_=6.3 is larger than the value of 1.0 expected for a statistical behaviour (see Supplementary Methods for the calculation of the statistical value)^31^,^32^, pointing to a weak positive cooperativity for the formation of the dimer. Also of interest is the value of *β*_1Er_, which is larger than other stability constants measured for the coordination of fluoride anions to similar Ln complexes^29^,^33^ (log *β*_1Eu_=4.6 was measured for pyridine containing cyclen analogue taking water concentration into account)^34^. The details of the calculated concentrations of the species formed during the titration and the recalculated ultraviolet–visible absorption spectra of the species are shown in Supplementary Figs 17 and 18.

Finally, the association was also monitored by ^1^H-NMR spectroscopy (Supplementary Fig. 19). As previously stated, the ^1^H-NMR spectrum of the Er complex in D_2_O is composed of 16 broad peaks in the +110/−110 p.p.m. region. Addition of fluoride anions up to 0.5 equivalent immediately resulted in the observation of a second set of 16 signals, the two sets coexisting for F/Er ratios smaller than 0.5. This number of peaks is in agreement with the formation of a dimeric complex with *D*_2_ symmetry and the observed variations of the chemical shifts are perfectly corresponding to the observations of Faulkner and co-workers^35^ with a change of magnetic anisotropy associated to an anisotropic electronic distribution from prolate to oblate upon fluoride binding. For excess larger than 0.5 equivalent, only minor changes could be observed for the shapes of the signals. In the presence of very large excesses (>5 equivalent), a broadening of the peaks could be noticed. This observation may be related to very minor changes in the coordination of the ligand around Er in the [(ErL)_2_F]^+^ dimer compared with the [ErLF] monomer as expected on the basis of density functional theory (DFT) modelling (see below). Unfortunately, attempts to follow the titration by ^19^F-NMR proved unsuccessful. The strong paramagnetic contribution of the Er cation with the short Er–F distances in the complexes probably resulted in too broad peaks that we could not observe.

### DFT modelling of the dimer

**Figure 5:**
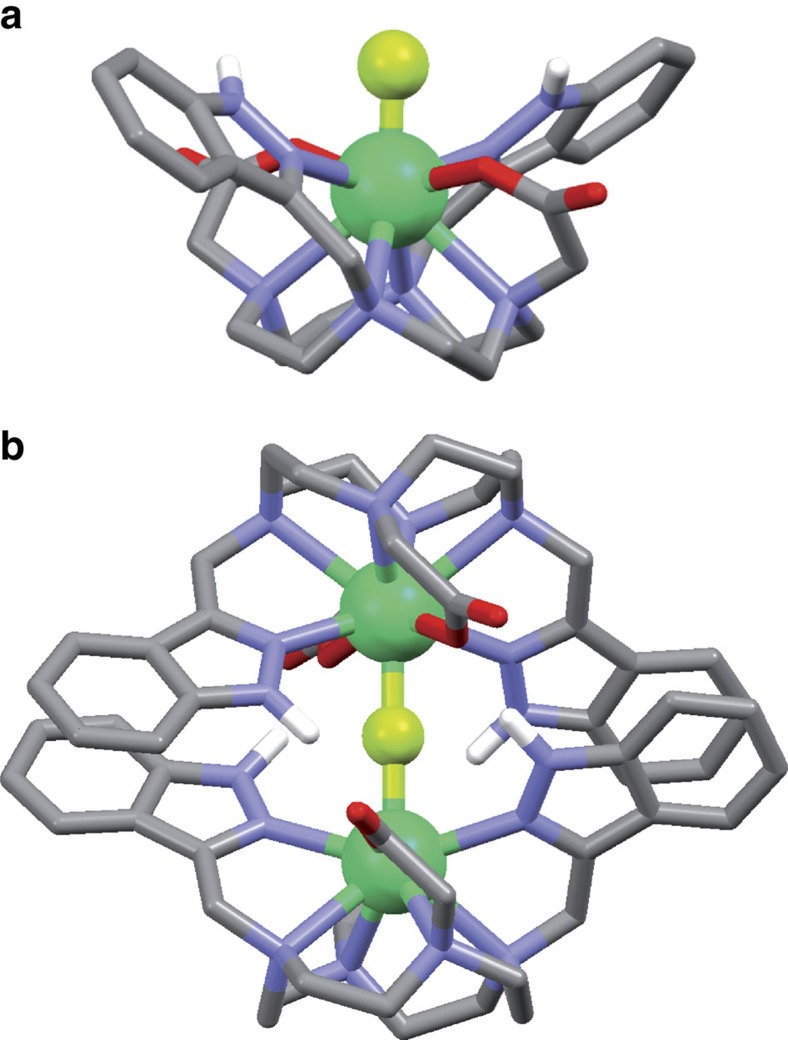
DFT modelling. The [ErLF] monomer (**a**) and the [(ErL)_2_F]^+^ dimer (**b**) were modelled by DFT calculations and revealed the presence of strong *π*–*π* stacking interactions between indazolyl groups in the dimer, together with four hydrogen bonding interactions which, with the formation of Er-F-Er bridge, stabilize the supramolecular dimeric assembly.

In the absence of available X-ray crystal structure, we turned our attention to DFT calculations for modelling the structure of the [ErLF] monomer and [(ErL)_2_F]^+^dimer (Fig. 5). DFT calculations performed at the M06/LCRECP/6-31G(d,p) level provided an optimized structure for [(ErL)_2_F]^+^ containing a linear Er-F-Er unit with a Er-F distance of 2.242 Å and a Er···Er distance of 4.484 Å. The Er-F distance is slightly shorter in the monomeric [ErLF] entity (2.104 Å). The dimeric structure is supported by *π*–*π* stacking interactions involving the indazolyl groups of the two (ErL) entities (distance between centroids 3.891 Å) and hydrogen bonds established between the NH groups of one indazolyl groups of a ErL entity and the oxygen atoms of the carboxylate groups coordinated to the second Er metal ion (N···O 2.751 Å, N-H···O 1.745 Å, N-H···O 162.4°). It is anticipated that the formation of hydrogen bonds and stacking interactions are at the origin of the positive cooperativity observed for the formation of the dimer during the ultraviolet–visible titration experiment (*vide supra*).

## Discussion

**Figure 6:**
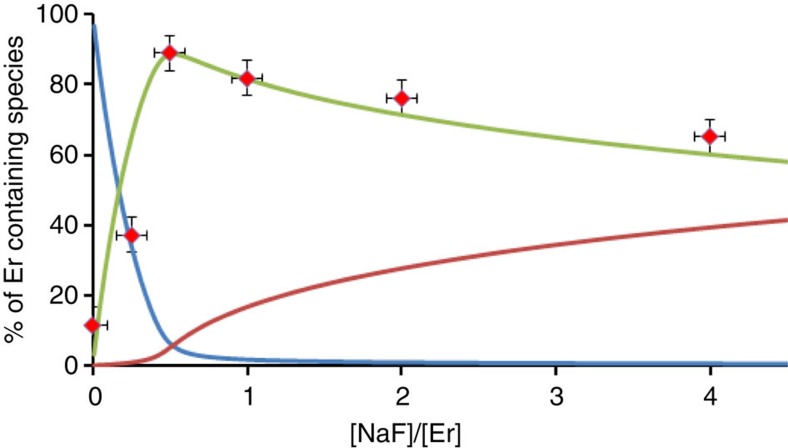
Speciation in solution. The relative evolution of the percentages of the species present in solution ([ErL(D_2_O)]^+^, blue; [(ErL)_2_F]^+^, green; [ErLF], red) can be recalculated from the knowledge of the stability constants and were compared with UC emission intensity (integrated from 505 to 580 nm, red squares) normalized on the concentration at 0.5 equivalent (errors correspond to those of a Poisson's law for the intensity and to ±5% for the relative concentrations). The speciation revealed a marked correlation between the UC intensity and the presence of the Er dimer.

Based on the stability constants determined above, it was possible to calculate the amounts of the three different species present in D_2_O solution, that is, [ErL(D_2_O)]^+^, [(ErL)_2_F]^+^ and [ErLF], as a function of added F^−^. Figure 6 represents the calculated concentrations of the different species as a function of the [F^−^]/[Er] concentration ratio. In the same graph, the evolution of the integrated intensity of the peak at 550 nm was reported, arbitrarily normalizing the emitted intensity at half an equivalent of added fluoride. The comparison clearly showed that the largest UC intensity increase is directly related to the formation of the dimer content. Interestingly, for larger amounts of fluoride for which the [ErLF] species is formed, the intensity decreased, pointing to a weaker UC process in the [ErLF] monomeric complex. It is expected that the coordination of fluoride anions in the monomeric complex will improve the metal-centred luminescence quantum yield by displacing the non-radiative quenching processes associated to OD oscillators of the coordinated D_2_O molecule oscillators of the coordinated heavy water molecule as observed by conventional fluorescence (Fig. 2b,c)^12^,^36^. This observation emphasizes the improvement of the UC process for the dimer compared with the two monomers, [ErL(D_2_O)] and [ErLF].

**Figure 7:**
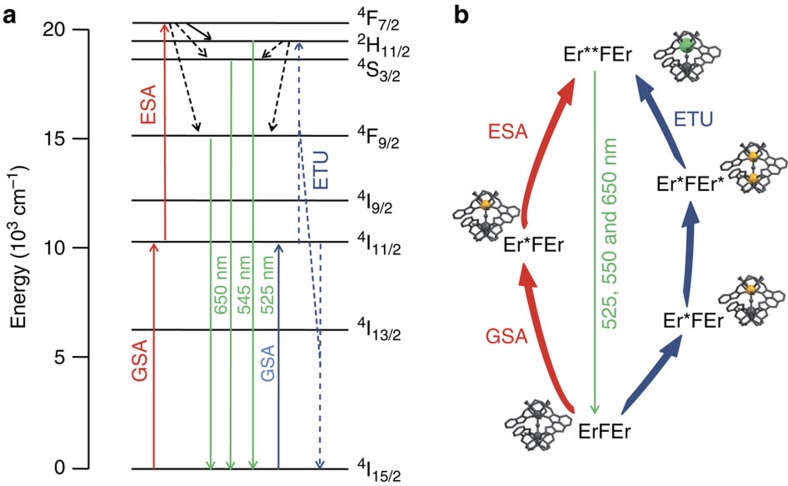
Energy level diagram and mechanisms of the UC process. (**a**) The different mechanisms (ETU in blue and excited state absorption (ESA) in red) for the UC process are presented in the energy level diagram of the Er ion. In both cases, the first step is the excitation to the ^4^I_11/2_ level upon excitation at 980 nm. For ESA, the erbium ion in the excited state absorbs a second photon to reach the upper levels and relaxation to the ^2^H_11/2_, ^4^S_3/2_ and ^4^F_9/2_ precedes the emission of the UC signal (green arrows). For ETU, the higher excited state is reached by energy transfer from the second Er atom itself in the ^4^I_11/2_ excited state. (**b**) Schematic representations of the two mechanisms (the vertical positions are not representative of the energy levels of the different excited states).

Considering first-order processes, Fig. 7 summarizes the two possible mechanisms responsible of the UC process in the dimer. For both mechanisms, the first step is a ground-state absorption resulting in the formation of an [Er*FEr] excited state with population of the ^4^I_11/2_ level of one Er atom. In the case of an ESA (red arrows), the absorption of a second photon by the excited Er atom led to population of the ^4^F_7/2_ level, [Er**FEr] and relaxation to the ^2^H_11/2_, ^4^S_3/2_ and ^4^F_9/2_ levels results in emission at 525, 550 and 650 nm, respectively. For the second process, the ETU (blue arrows), the ground-state absorption leading to [Er*FEr] is followed by the absorption of a second photon by the second Er atom, so that both Er atoms were in the ^4^I_11/2_ excited state, forming the [Er*FEr*] excited state. An intramolecular energy transfer from one Er to the other relaxed one Er to the ground state, whereas the second was promoted to a higher energy level such as ^4^F_7/2_, ^2^H_11/2_ or ^4^S_3/2_, depending on whether the transfer process is a phonon-assisted one or not. Relaxation to the ground state also led to emission at 525, 550 and 650 nm.

A major difference in the ESA and ETU processes can be found in the risetime of the UC emitted signal. As ESA arise from two absorption phenomena, the risetime is very rapid. In contrast, the ETU process is slowed by the rate of energy transfer from the intermediate excited state and may lead to an observable risetime. Recording the risetime of the emission at 545 nm (^4^S_3/2_→^4^I_15/2_) upon pulsed laser excitation at 980 nm (Supplementary Fig. 20) showed a *ca* 5 μs risetime in the emission, pointing to an ETU mechanism in the Er dimer.

The relationship between the UC efficiency and the concentration of the species in solution points to two observations; the efficiency is directly related to the presence of the Er dimer and decreases as the concentration of [ErLF] monomer increases. Both observations are in favour of an ETU mechanism. If it proceeded via the ESA mechanism, the efficiency of the UC process should not decrease for larger concentrations of [ErLF] monomer as the metal-centred luminescence quantum yield of the monomer was found to be almost similar to that of the dimer (Fig. 2). If one hypothesizes that the crystal field splitting and the transition probabilities of Er are similar for the monomer and the dimer (DFT models point to similar coordination environment around Er in the monomer and the dimer), the UC efficiency should also be the same, which is not the case. Although the UC signals observed for the monomers are probably related to an ESA mechanism, as observed by Faulkner and co-workers^19^, the 7.7-fold increase of the UC efficiency associated with the formation of the dimer can hardly be explained only by the ESA mechanism, considering that the one photon luminescence quantum yield only increased by a factor 1.6. These observations, together with the evidence of a rising domain in the UC emitted signal, are fully consistent with an ETU mechanism, favoured by the spatial proximity of the two Er centres, as it has been observed in Er doped solids^37^.

The coordination of fluoride anions with the [ErL(H_2_O)]^+^ complex resulted in the self-assembly of two complexes around a single fluoride anion. The assembly is characterized by a positive cooperative effect resulting from the formation of hydrogen bonds and aromatic stacking interactions. DFT models of the dimer showed that the intermetallic distance is very short (4.48 Å). In a D_2_O solution at room temperature, excitation of the Er complex resulted in the observation of three UC emission bands at 525, 550 and 650 nm, respectively, attributed to the Er centred ^2^H_11/2_→ ^4^I_15/2_, ^4^S_3/2_→^4^I_15/2_ and ^4^F_9/2_→^4^I_15/2_ transitions, upon excitation in the Er ^4^I_15/2_→^4^I_11/2_ absorption band at 980 nm. Noticeably, addition of fluoride anions to the solution is accompanied by a very large 7.7-fold increase of the UC emission attributed to the formation of the dimer in a D_2_O. These results unambiguously showed that UC can be achieved at the molecular and supramolecular level, in solution under ambient conditions, providing that the system is well-adapted. Although the observed UC may be related to an ESA mechanism in the monomeric complexes, the large increase observed for the dimer and the risetime observed for the UC emitted signal emphasize the importance of the association of the two cations of the dimer in the UC process and strongly indicate an excited state energy transfer mechanism. In this context, the very short intermetallic distance between the two Er cations is believed to be a crucial parameter.

These results are the first evidence of UC at the molecular level in deuterated aqueous solution at room temperature. We can expect that future developments on the structure and compositions of such complexes could lead to similar observations in non-deuterated water, hence opening a brand new window for luminescence tagging in numerous bio-analytical applications.

## Methods

### Materials and chemical analysis

Solvents and starting materials were purchased from Aldrich, Acros and Alfa Aesar and used without further purification. ^1^H and ^13^C NMR spectra were recorded on Bruker 500, 400 or 300 spectrometers. Chemical shifts are reported in p.p.m., with residual protonated solvent as internal reference^38^. Infrared spectra were recorded on a Perkin Elmer Spectrum One Spectrophotometer as solid samples and only the most significant absorption bands are given in cm^−1^. Elemental analyses and routine mass spectrometry analysis were carried out by the Service Commun d'Analyses of the University of Strasbourg. High-resolution electrospray ionisation-time of flight (ESI-TOF) mass spectra were recorded using a micro-TOF-Q Applied Biosystems QSTAR Elite spectrometer in the positive mode.

### Computational details

All calculations presented in this work were performed employing the Gaussian 09 package (Revision D.01)^39^. Full geometry optimizations of the [(ErL)_2_F]^+^ and [ErLF] systems were performed in aqueous solution employing DFT within the hybrid meta generalized gradient approximation (hybrid meta-GGA), with the M06 exchange-correlation functional^40^. In these calculations, we used the large-core quasirelativistic effective core potential of Dolg and co. and its associated [5s4p3d]-GTO valence basis set for Er^41^, whereas the ligand atoms were described by using the standard 6–31G(d,p) basis set. Solvent effects were included by using the CPCM variant of the polarizable continuum model, as implemented in Gaussian 09 (refs 42, 43).

### Spectroscopic characterizations

Spectroscopic measurements were performed with 10 × 10 mm^2^ quartz suprasil certified cells (Helma Analytics). Ultraviolet–visible absorption spectra were recorded on a Perkin Elmer Lambda 950 spectrometer. Steady-state emission spectra were recorded on a FLS920P fluorescence spectrometer (Edimburgh Instrument) equipped with a 450-W continuous wavelength Xe lamp (range from 230 to 900 nm), using Hamamatsu R928 (visible) or R5509-72 (Vis and NIR range) photomultipliers. For steady-state emission in the NIR range, high-pass filters at 850 nm were used to remove second-order artefacts. For UC emission spectra, a 980-nm continuous wavelength laser diode (5 W, Laserwave, Beijing, China) was used as the light source and emission in the visible was detected with a photomultiplier tube (PMT, R928 Hamamatsu). Power dependence of UC emission was measured under a focused excitation light with a spot size (average) of ≈0.07 cm^2^, and laser power increased step by step (0.9–2.3 W). A focus lens (focal length is 10 cm) collimating the laser light (diameter of light cross section is about 5 mm) was placed 10 cm in front of the 980 nm laser, and 5 cm before the aperture. A power metre (FieldMaxII-TO laser power meter and PM30 probe) was placed in a position close to where the cuvette would be to measure the power. Laser power is measured both before and after spectral measurement of solution samples with the power probe placed in the pre-set position. Considering the response time of power metre probe (2 s), laser power is measured after allowing the laser to stabilize for 30 s. The error of the laser power and the power metre is <1%, respectively. The UC excitation spectra and UC kinetic experiments were performed with an ultrafast laser system equipped with R928 Hamamatsu Photomultiplier Tube. Excitation light was generated by an infrared coherent pulsed laser (Ti:sappire compact laser system- 150 fs, with wavelength range 690–1,040 nm), excitation power at every point was calibrated to 750 mW and the UC emission intensity was monitored at 545 nm (^4^S_3/2_+^2^H_11/2_→^4^I_15/2_).

### Synthesis of the Er complex

[ErL(H_2_O)](NO_3_) was synthesized by adaptation of previously published methods^22^ using nitrate salts of Er instead of lanthanide chloride salts. Yield: 77%. ^1^H NMR (D_2_O, 400 MHz, 25 °C, Supplementary Figs 1 and 2): *δ* 110.16, 80.61, 45.98, 25.64, 21.47, 20.22, 15.50, 14.19, 12.12, 3.09, −1.52, −21.61, −82.97, −100.84, −110.54; infrared (attenuated total reflectance (ATR), Supplementary Fig. 3): 3,437 (br, m), 3,216 (br, m), 2,863 (w), 1,655 (m), 1,614 (s), 1,356 (s), 1,324 (s), 1,077 (m), 1,003 (w), 931 (w), 756 (m); ultraviolet–visible (H_2_O): *λ*_max_=294 nm; electrospray mass spectrometry (ES-MS) (ESI^+^, Supplementary Fig. 4): [ErL]^+^ calculated for C_28_H_34_ErN_8_O_4_, 714.19; found, 714.20; analysis (calculated, found for [(ErL)H_2_O](NO_3_).NaCl.4H_2_O (C_28_H_44_ClErN_9_NaO_12_)): C (36.38, 36.49), H (4.80, 4.42); N (13.64, 13.62).

### Data availability

The authors declare that all relevant data are available from the authors on request.

## Additional information

**How to cite this article:** Nonat, A. *et al.* Room temperature molecular up conversion in solution. *Nat. Commun.* 7:11978 doi: 10.1038/ncomms11978 (2016).

## Supplementary Material

Supplementary Information Supplementary Figures 1-20, Supplementary Methods and Supplementary References

## Figures and Tables

**Figure 1:**
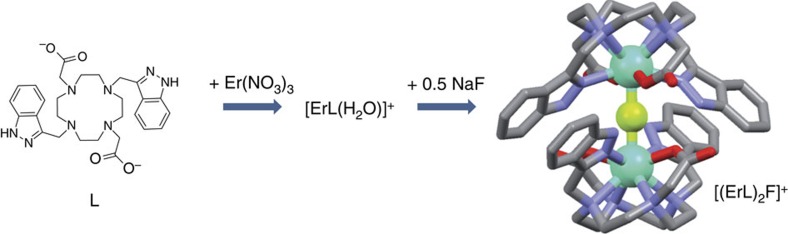
Formation of the supramolecular erbium dimer. The ligand L react with Er^3+^ salts in water to form an [ErL(H_2_O)]^+^ complex. In the presence of half an equivalent of fluoride anions two complexes form an association in which the F^−^ anion is sandwiched between the two complexes leading to the [(ErL)_2_F]^+^ dimer. The association is strengthened by two intercomplex stacking interactions and four hydrogen bonds.
