# Supplementary material for: Room temperature molecular up conversion in solution
Source: Nat Commun. 2016 Jun 15;7:11978. doi: 10.1038/ncomms11978 (PMC4912637; doi:10.1038/ncomms11978)
Supplement: Supplementary Information — Supplementary Figures 1-20, Supplementary Methods and Supplementary References [file ncomms11978-s1.pdf]

Supplementary Information

Supplementary figures.

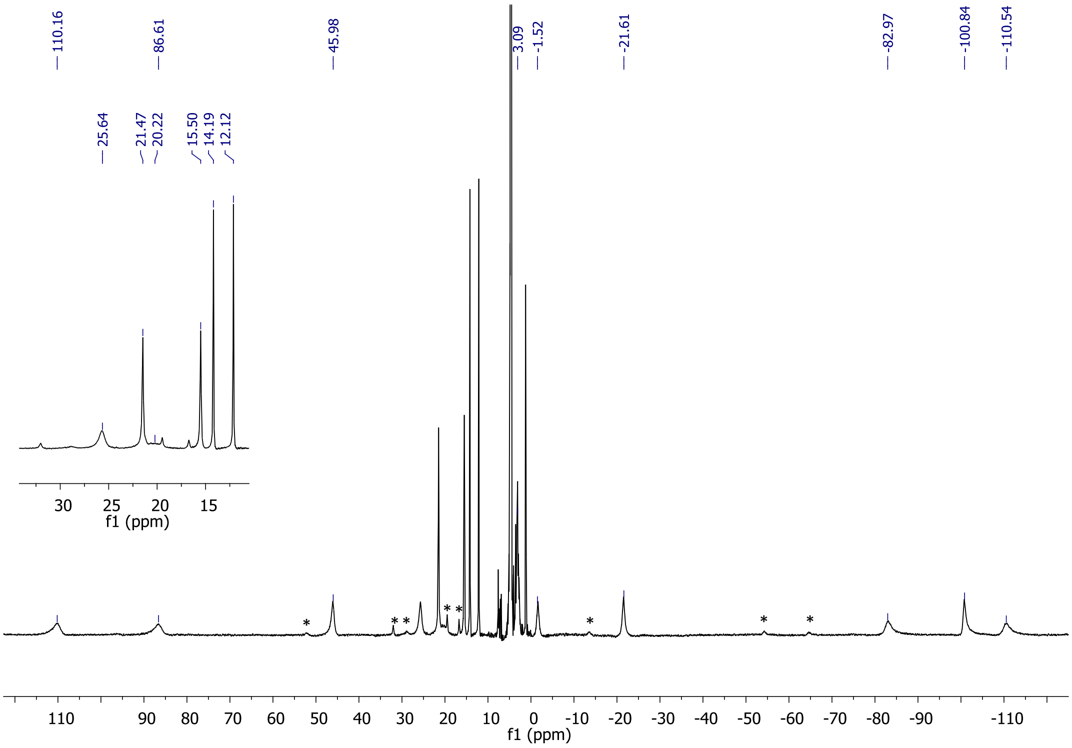

Supplementary Fig 1. ^{1}H-NMR of the Er complex in D_2O (400 MHz, 298 K). Peaks denoted with a star correspond to the presence of a minor amount (<6%) of a paramagnetic complex attributed to the TSAP isomer.

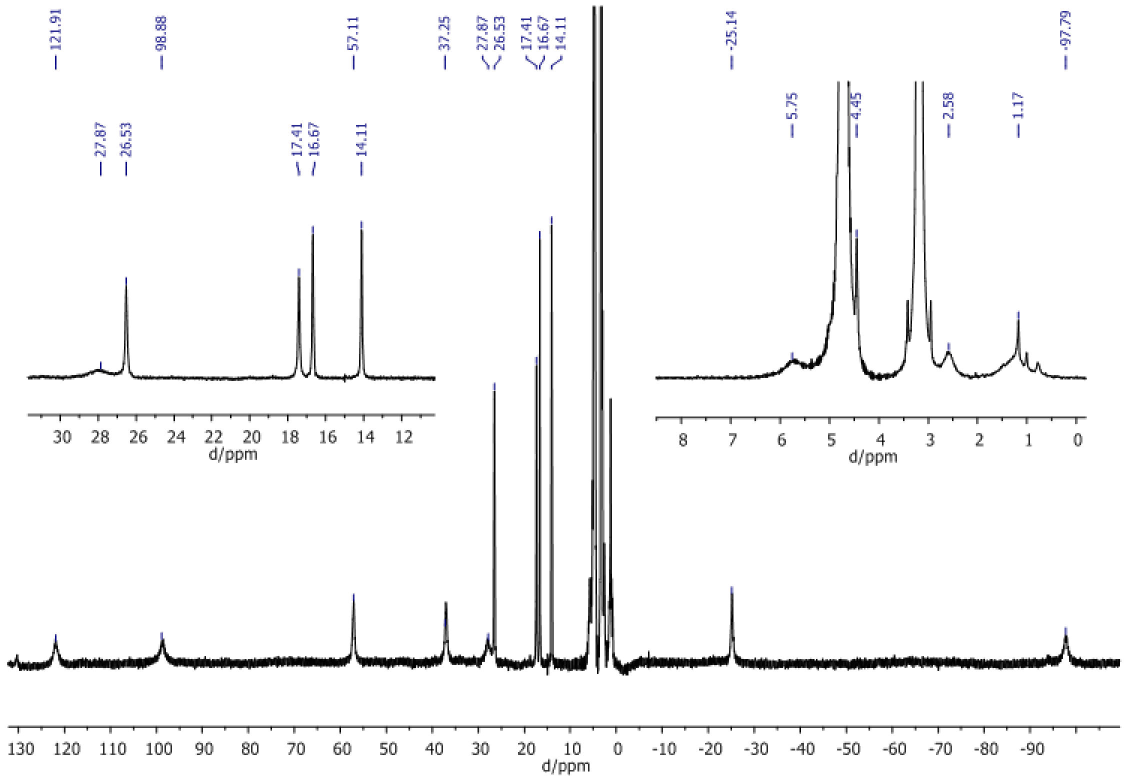

Supplementary Fig 2. ^{1}H-NMR of $[ErL(H_2O)](NO_3)$ in CD_3OD (300 MHz, 298 K).

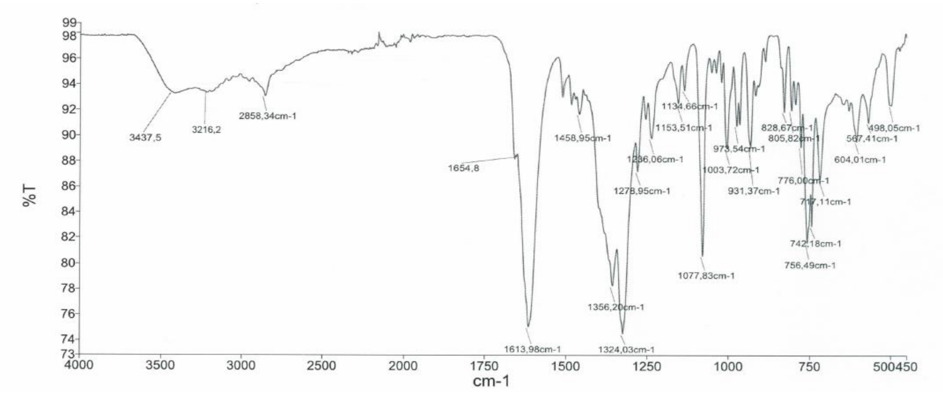

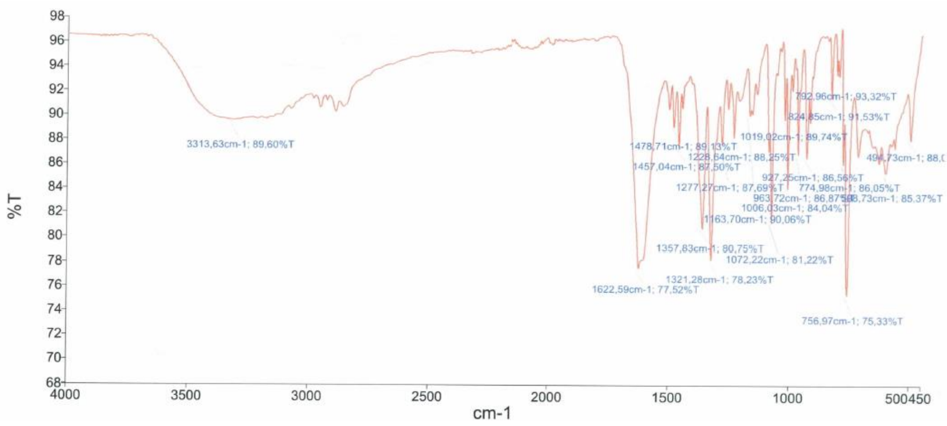

Supplementary Fig 3. IR spectrum (ATR) of [ErL(H_2O)](NO_3) (top) and of [EuL(H_2O)]Cl (bottom) showing the appearance of new bands at 1655 and 1360 cm^{-1} attributed to vibrations of the nitrate anion.

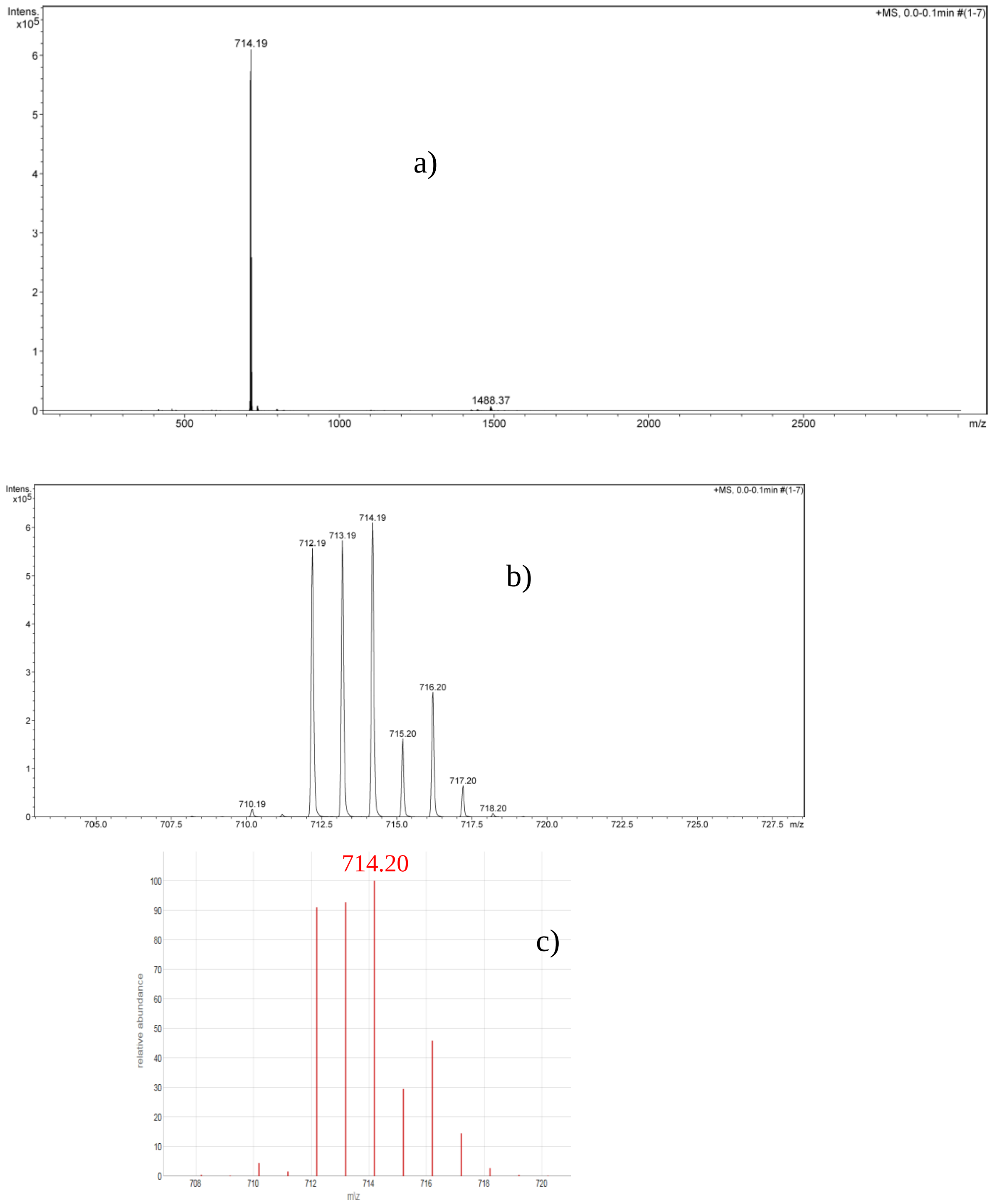

Supplementary Fig 4.(a) ES/MS of [ErL(H_2O)](NO_3) in water; (b) enlargement showing the isotopic distribution of the peak with maximum at 714.19 *m/z* units; and (c) calculated isotopic distribution for $[ErL]^+$ ($C_{28}H_{34}ErN_8O_4$)$^+$.

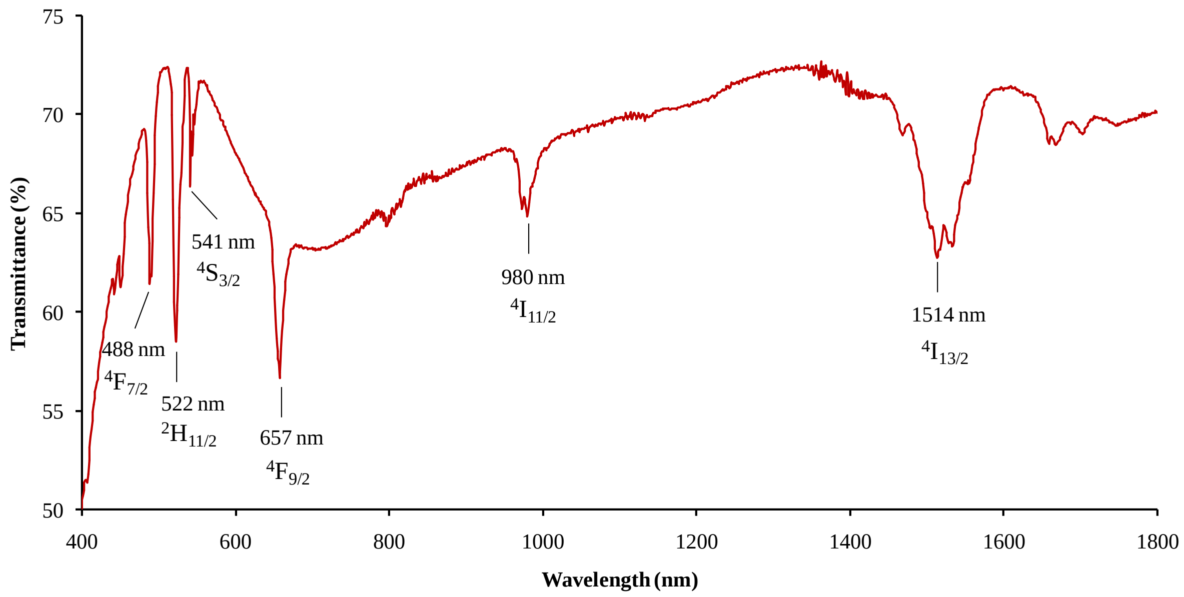

Supplementary Fig 5. Transmittance spectrum of the [ErL(H_2O)] complex in the solid state. Transmittance spectra was measured using a UV-Vis-NIR Perkin Elmer Lambda 950 fitted with a Prying Mantis^{TM} from Harrick. Detection in the visible range (250-860 nm) was performed with a PMT and by using a PbS detector in the NIR region (860-1800 nm).

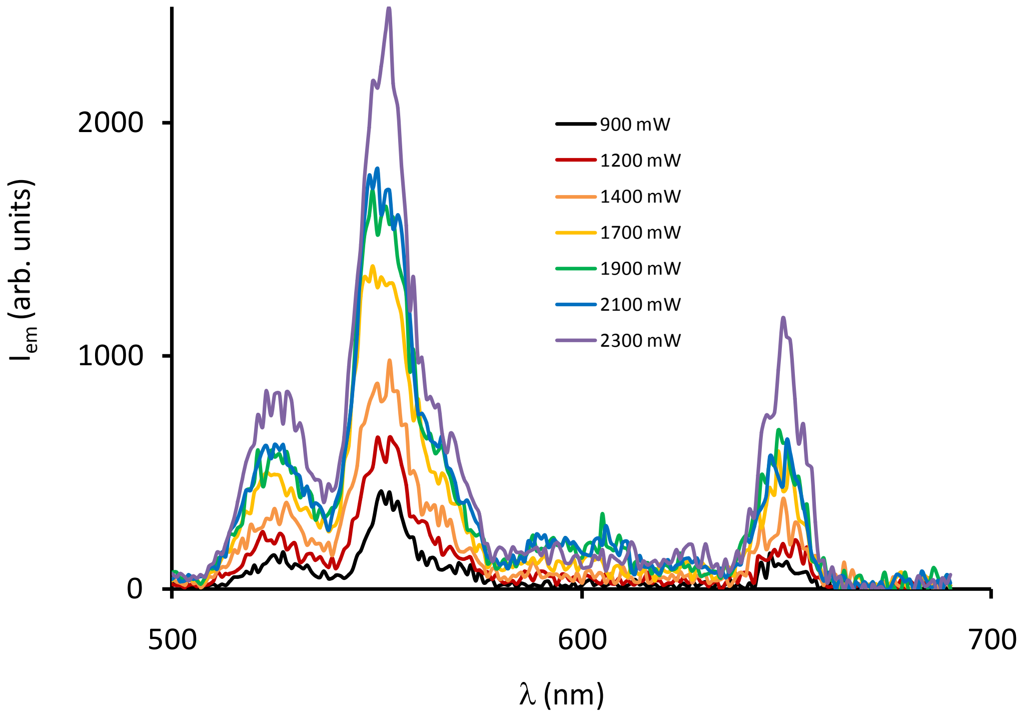

Supplementary Fig 6. UC spectra of a 1 mM solution of the Er complex in D_2O containing 0.5 equivalent of NaF as a function of the power of the incident beam at 980 nm.

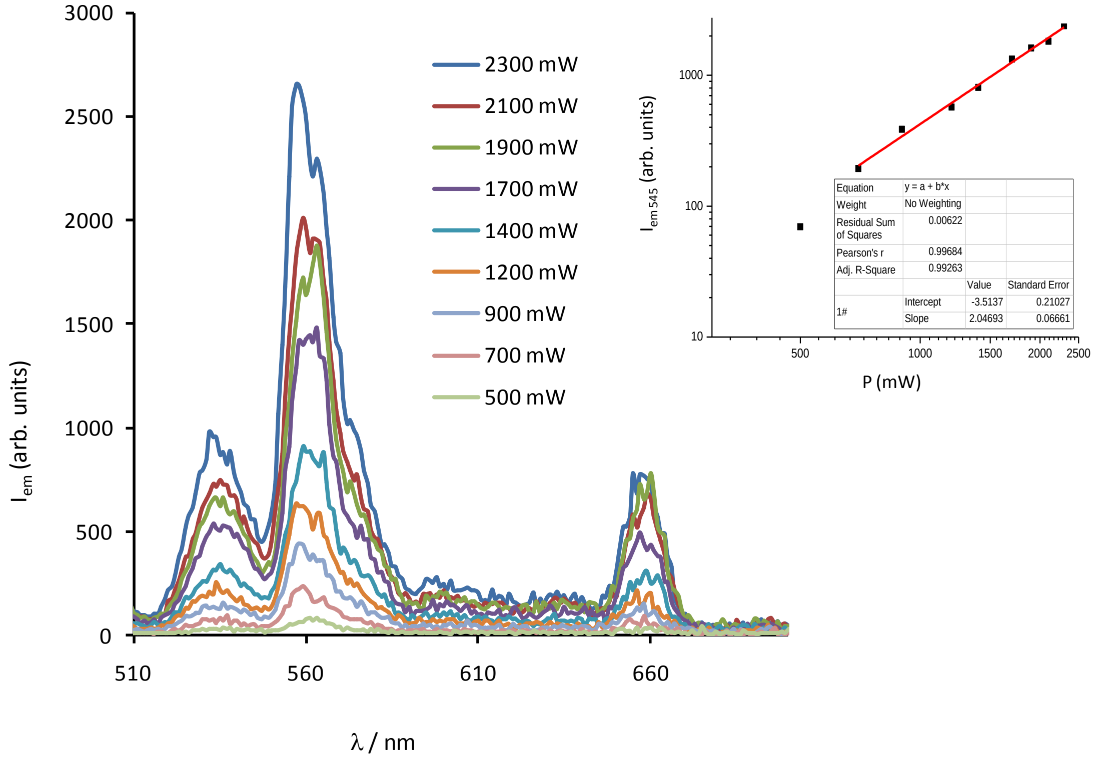

Supplementary Fig 6 bis. UC spectra of a 1 mM solution of the Er complex in D_2O containing 0.5 equivalent of NaF as a function of the power of the incident beam at 980 nm. Inset: LogI/LogP and its linear fitting (third experiment).

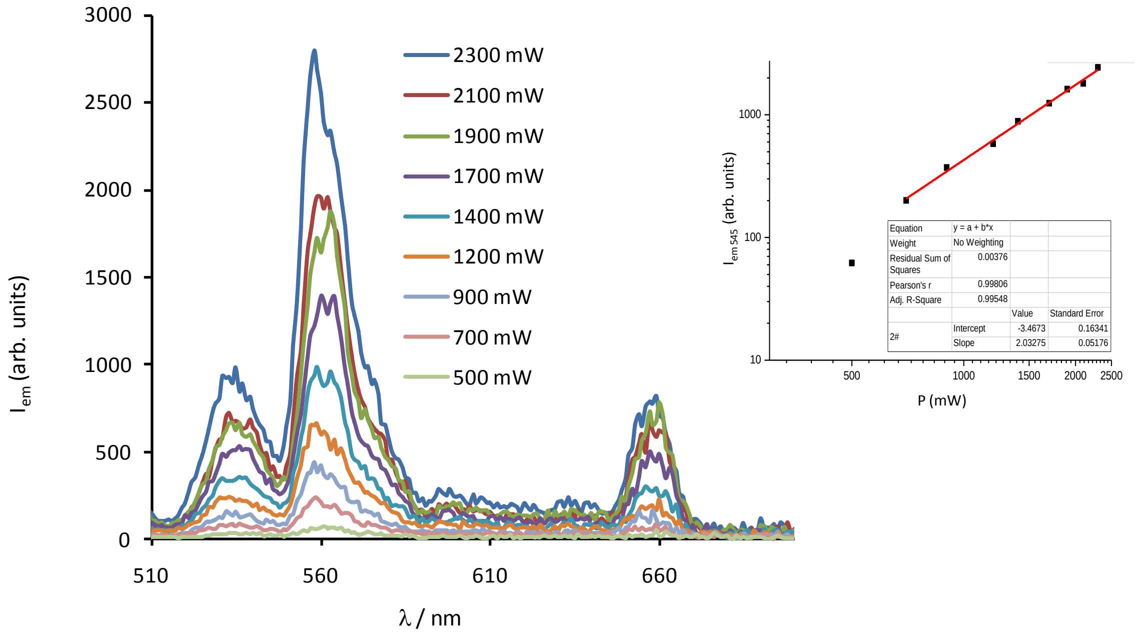

Supplementary Fig 6 ter. UC spectra of a 1 mM solution of the Er complex in D_2O containing 0.5 equivalent of NaF as a function of the power of the incident beam at 980 nm. Inset: LogI/LogP and its linear fitting (fourth experiment).

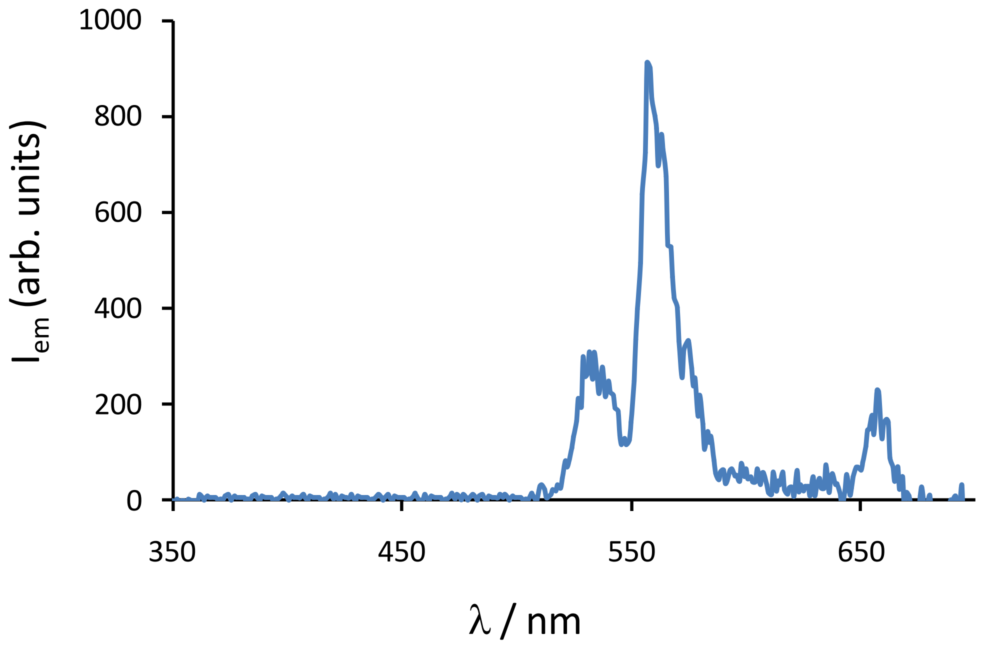

Supplementary Fig 7. Full (350 to 750 nm) UC spectrum of a 200 µM solution of the Er complex in D_2O containing 0.5 equivalent of NaF (λ_{exc} = 980 nm).

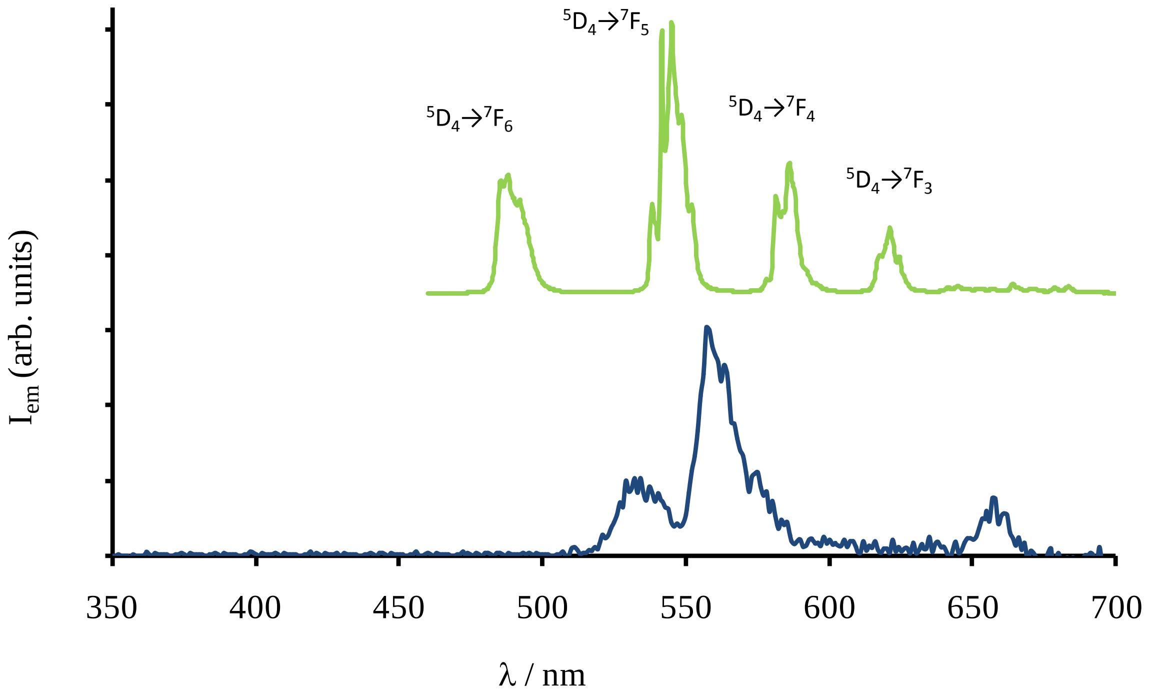

Supplementary Fig 8. Comparison of the UC spectrum of $[ErL(D_2O)](NO_3)$ in the presence of one equivalent of NaF in D_2O (λ_{exc} = 980 nm, bottom) and emission spectrum of $[TbL(H_2O)]Cl$ (λ_{exc} = 326 nm top).

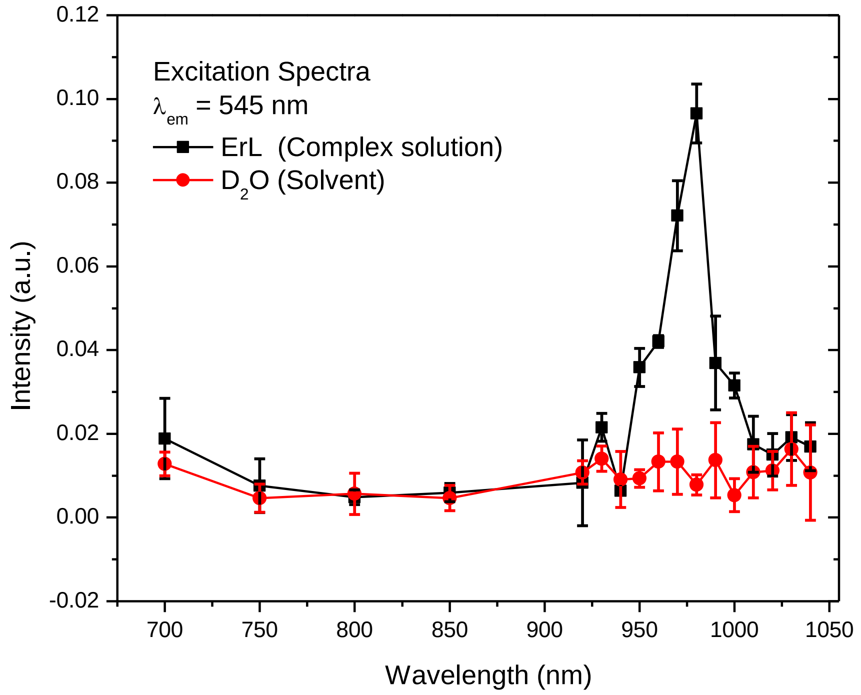

Supplementary Fig 9. UC excitation spectra of ErL complex in the presence of half an equivalent of fluoride and D_2O solvent (λ_{em} = 545 nm).

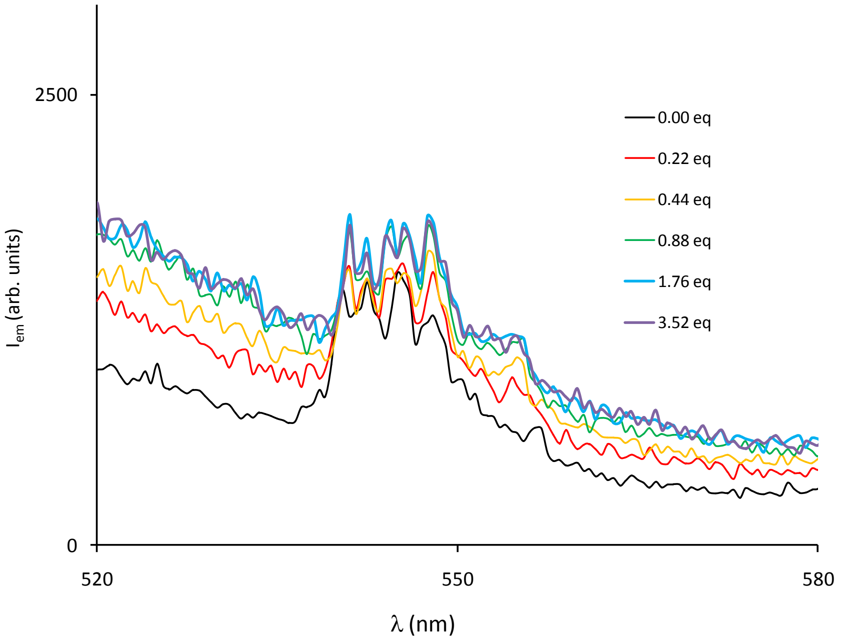

Supplementary Fig 10. Evolution of the emission intensity (λ_{exc} = 294 nm) of the $^4S_{3/2} \rightarrow {}^4I_{15/2}$ transition of Er (D_2O, [c] = 0.22 mM) upon addition of NaF.

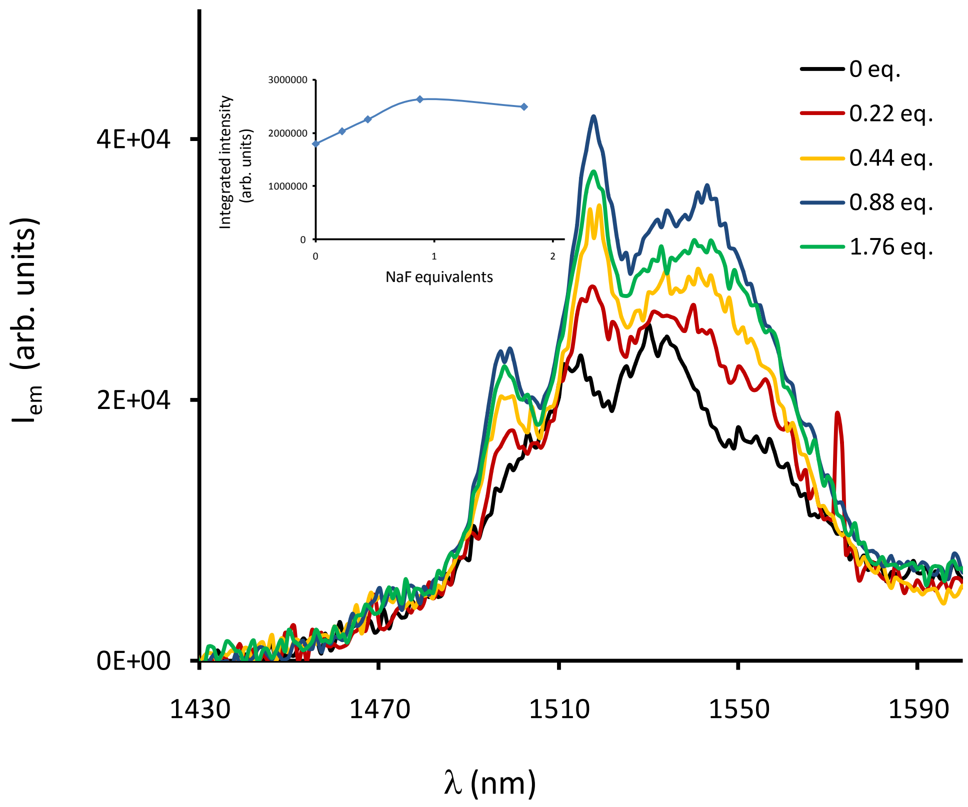

Supplementary Fig 11. Evolution of the emission intensity (λ_{exc} = 980 nm, P = 2 W) of the $^4I_{13/2} \rightarrow {}^4I_{15/2}$ transitions of Er (D_2O, [c] = 0.22 mM) upon addition of NaF. Inset: Integrated intensity of the $^4I_{13/2} \rightarrow {}^4I_{15/2}$ transition from 1450 to 1600 nm.

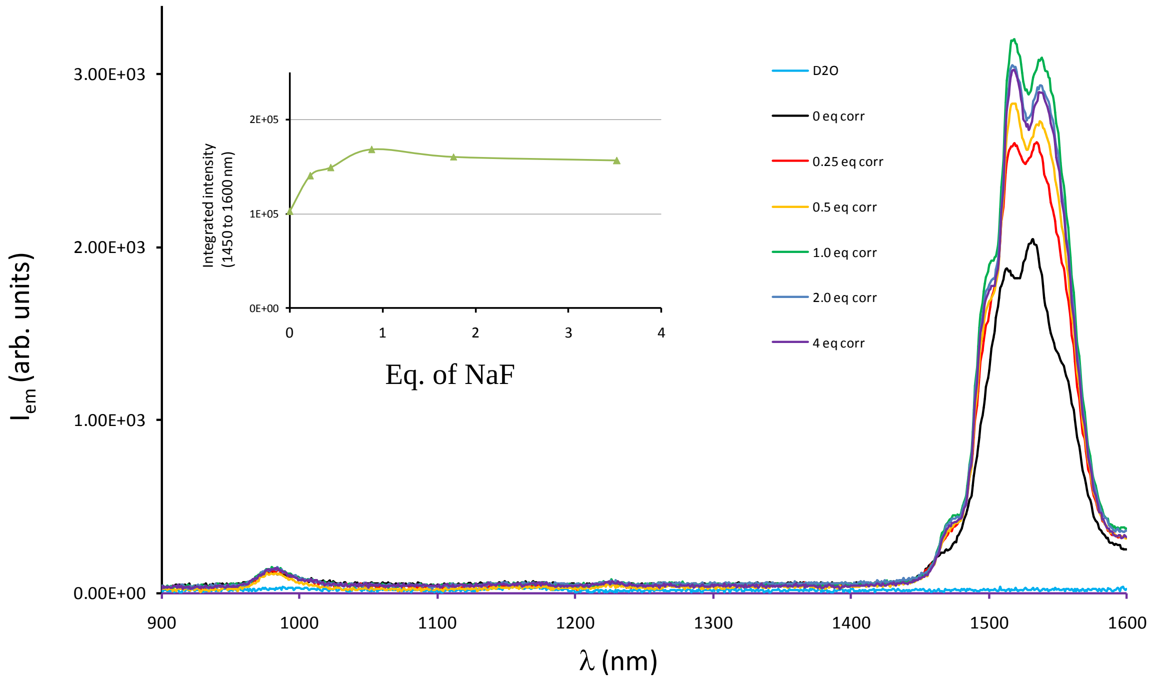

Supplementary Fig 12. Evolution of the emission intensity (λ_{exc} = 294 nm) of the $^4I_{11/2} \rightarrow$ $^4I_{15/2}$ (980 nm) and $^4I_{13/2} \rightarrow$ $^4I_{15/2}$ (*ca* 1530 nm) transitions of Er (D_2O, [c] = 0.22 mM) upon addition of NaF. Inset: Integrated intensity of the $^4I_{13/2} \rightarrow$ $^4I_{15/2}$ transition from 1450 to 1600 nm.

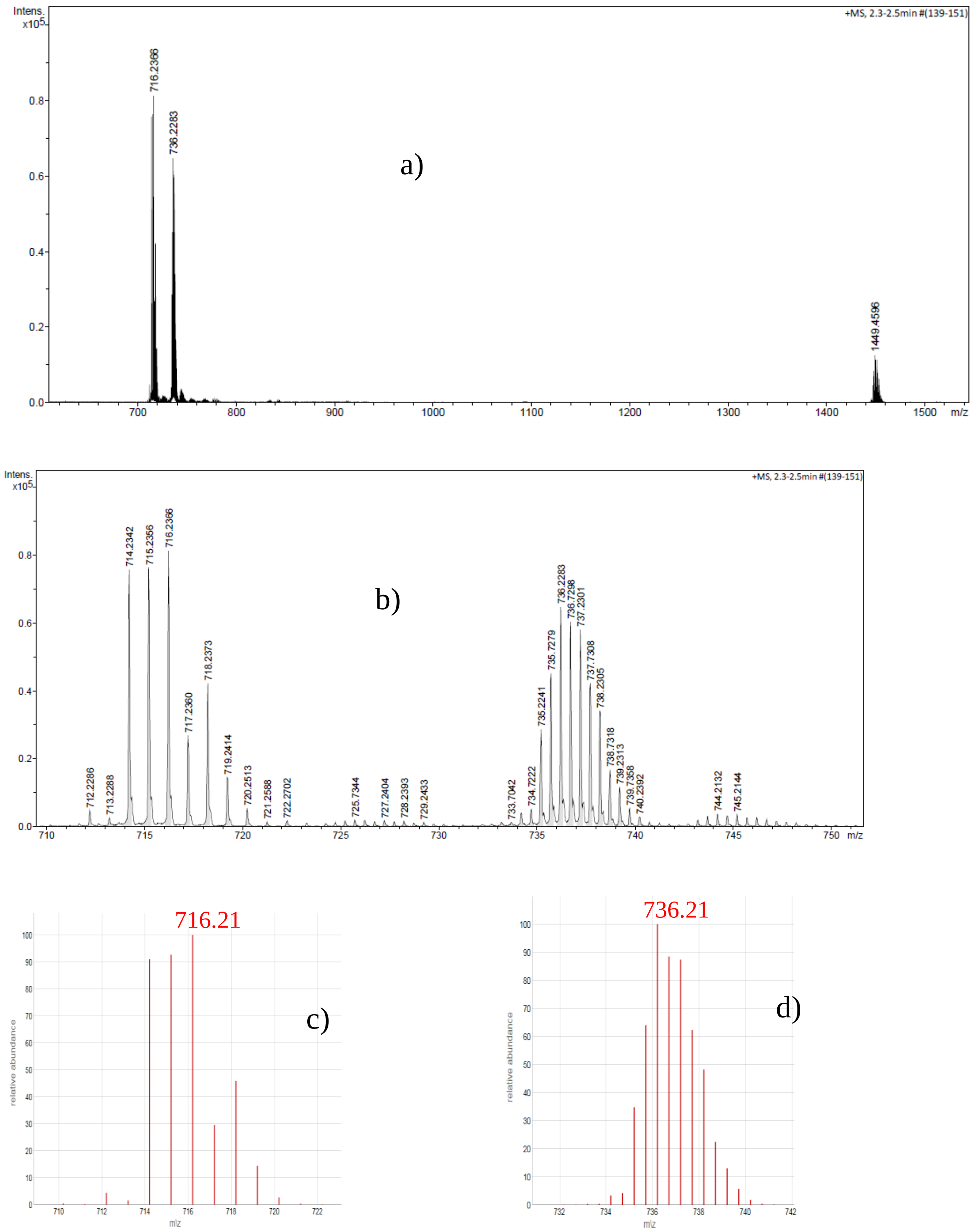

Supplementary Fig 13. (a) ES/MS of a D_2O solution of [Er(H_2O)](NO_3) in presence of 0.5 equivalent of NaF; (b) enlargement of the region from 710 to 750 showing the massif of $[ErL]^+$ pointing at 716.21 *m/z* and the $[(ErL)_2F+Na]^{2+}$ dimer at 736.21 *m/z*; (c) isotopic distribution calculated for $[ErL]^+$ ($C_{28}H_{32}D_2ErN_8O_4$); and (d) isotopic distribution calculated for $[(ErL)_2F+Na]^{2+}$ ($C_{56}H_{64}D_4Er_2FNaN_{16}O_8$)

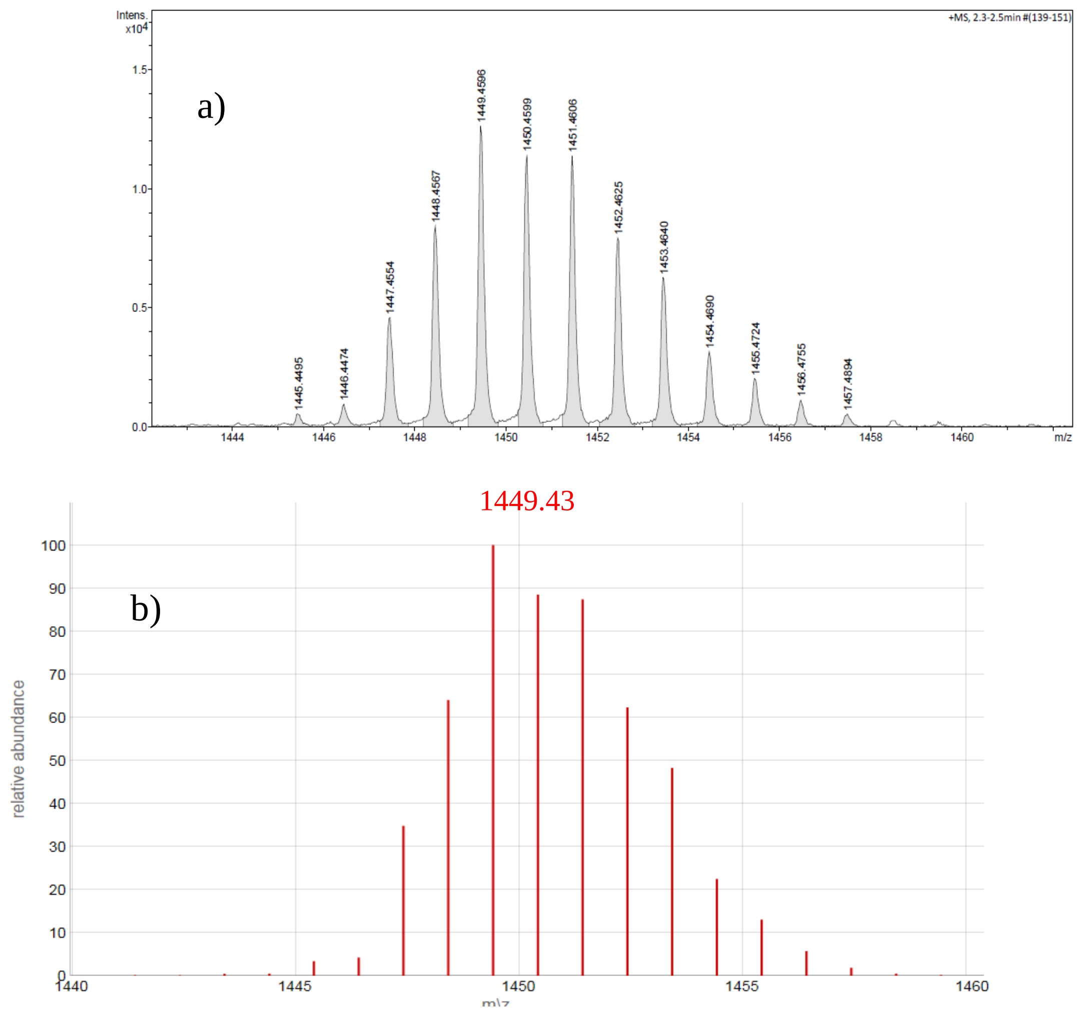

Supplementary Fig 14. (a) Enlargement of the region of the spectrum of a D_2O solution of $[Er(H_2O)](NO_3)$ in presence of 0.5 equivalent of NaF showing the massif for $[(ErL)_2F]^+$; and (b) isotopic distribution calculated for $[(ErL)_2F]^+$ ($C_{56}H_{64}D_4Er_2FN_{16}O_8$).

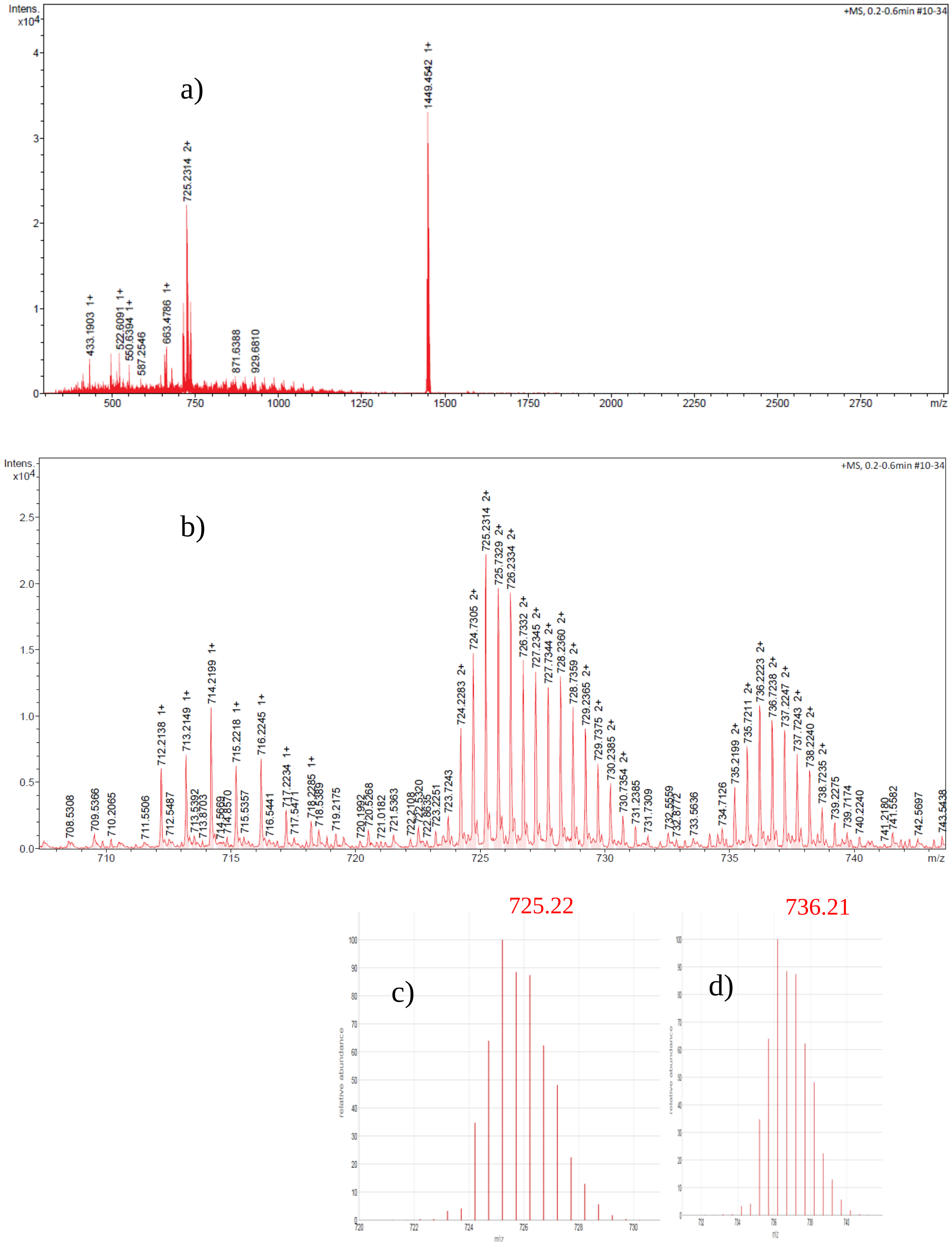

Supplementary Fig 15. (a) ES/MS of a D_2O solution of [Er(H_2O)](NO_3) in presence of 1.0 equivalent of NaF; (b) enlargement of the region from 708 to 745 showing the massifs of $[(ErL)_2F + H]^{2+}$ pointing at 725.23 *m/z* and the $[(ErL)_2F{+}Na]^{2+}$ dimer at 736.21 *m/z*; (c) isotopic distribution calculated for $[(ErL)_2F + H]^{2+}$ ($C_{56}H_{65}D_4Er_2FN_{16}O_8$); and (d) isotopic distribution calculated for $[(ErL)_2F{+}Na]^{2+}$ ($C_{56}H_{64}D_4Er_2FNaN_{16}O_8$).

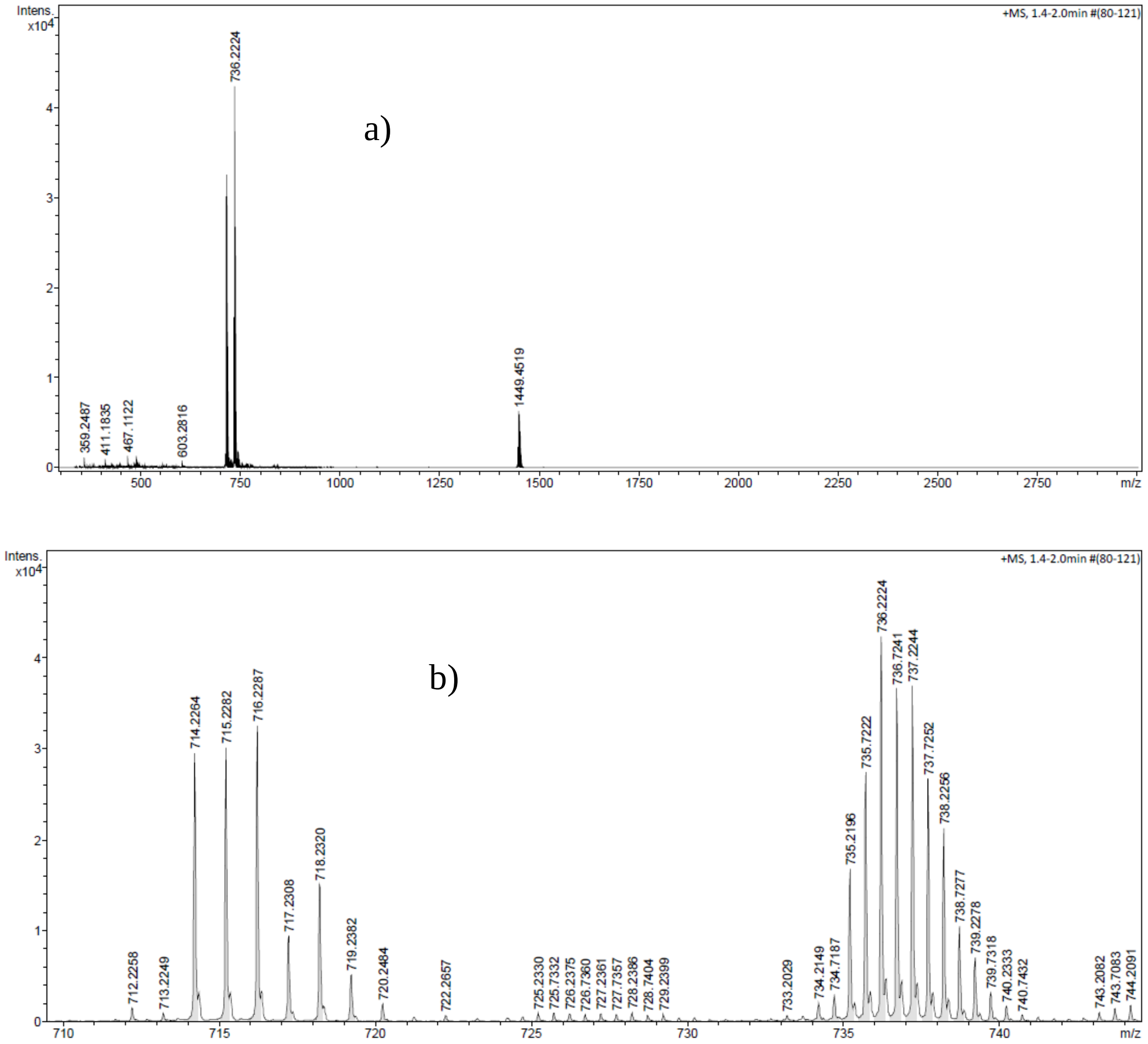

Supplementary Fig 16. (a) ES/MS of a D_2O solution of $[Er(H_2O)](NO_3)$ in presence of 2.0 equivalent of NaF; (b) enlargement of the region from 708 to 745 showing the massives of $[ErL]^+$ pointing at 716.22 *m/z* and the $[(ErL)_2F+Na]^{2+}$ dimer at 736.21 *m/z*. Isotopic distributions calculated for the $[(ErL)_2F+Na]^{2+}$ dimer at 736.21 *m/z*, for the $[ErL]^+$ complex at 716.22 *m/z*, and for the $[(ErL)_2F]^+$ dimer at 1449.45 *m/z* can be found respectively in Supplementary Fig 15d, 13c and 14b).

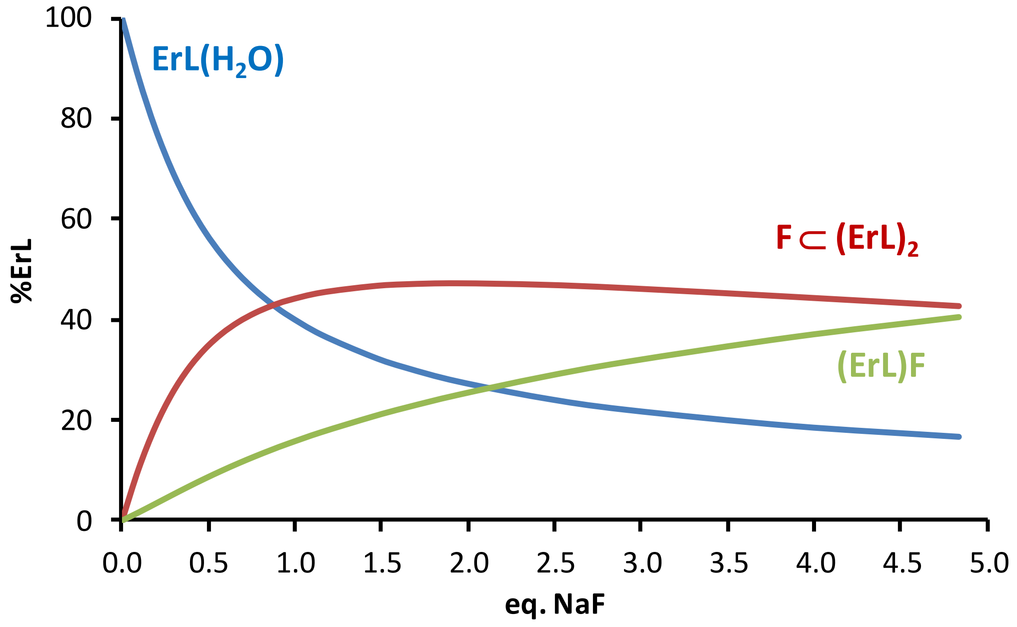

Supplementary Fig 17. Evolution of the concentrations of the species formed upon titration of a 3.15×10^{-5} M solution of $[ErL(H_2O)]Cl$ upon addition of NaF.

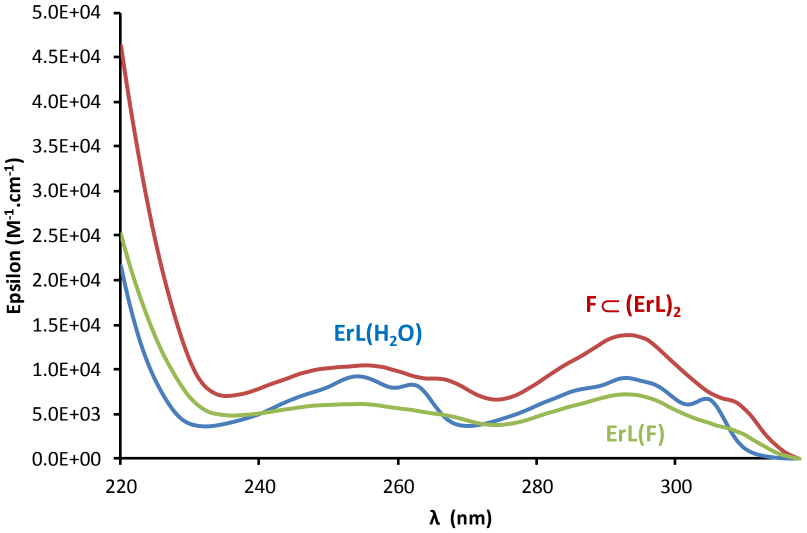

Supplementary Fig 18. Calculated spectra of the species formed during the titration of a 3.15×10^{-5} $M^{-1}.cm^{-1}$ solution of [ErL(H_2O)](NO_3) upon addition of NaF.

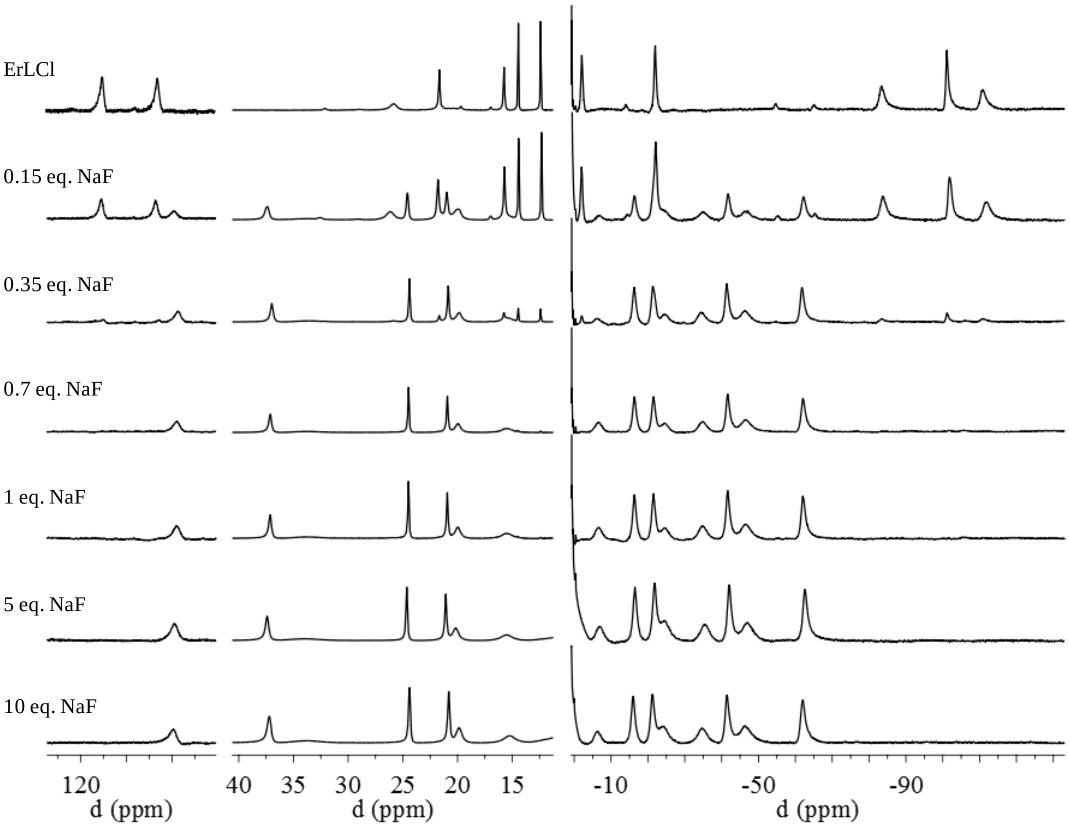

Supplementary Fig 19. ^{1}H-NMR titration of the Er complex by addition of NaF in D_2O (298 K, 400 MHz).

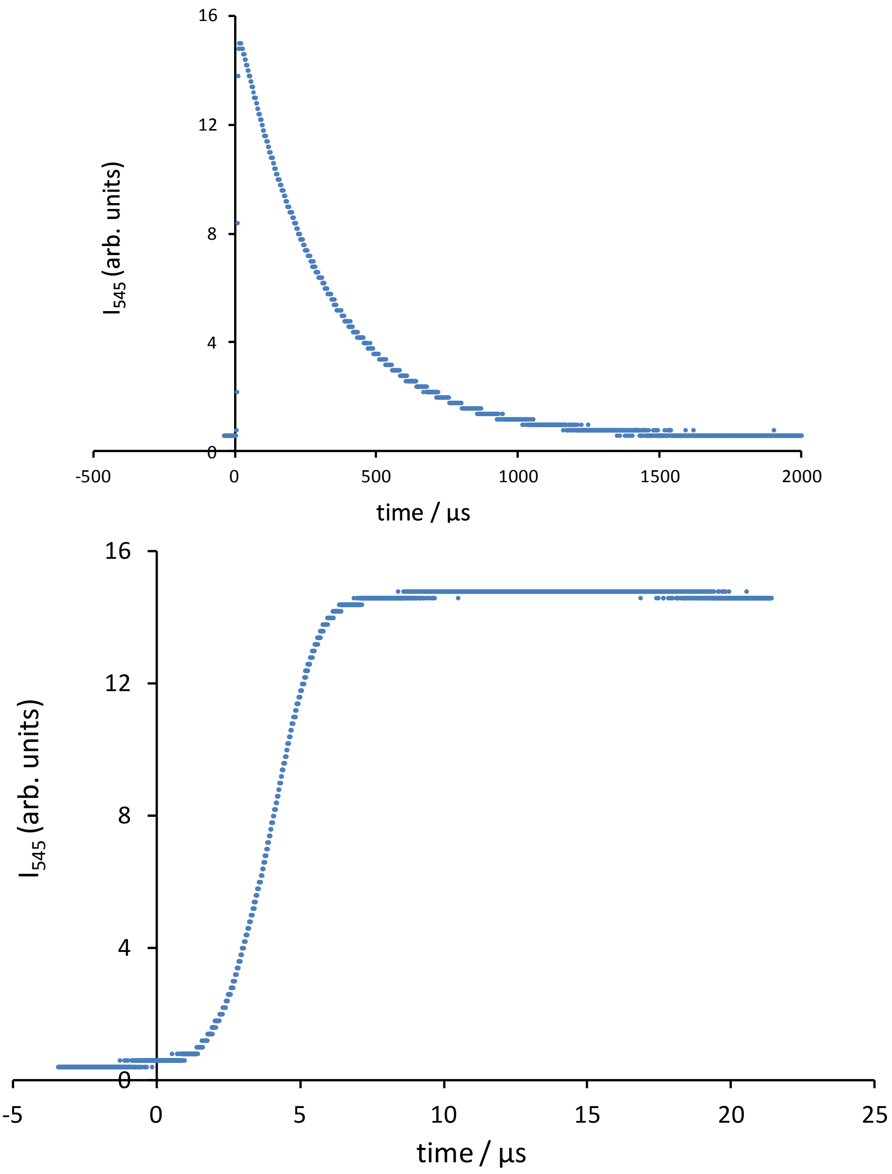

Supplementary Fig 20. Top) Evolution of the emission at 545 nm ($^4S_{3/2} \rightarrow {}^4I_{15/2}$ transition of Er) upon pulsed excitation with a 10 ns pulsed laser at 980 nm. Bottom) Enlargement of the rising part of the signal.

Supplementary Methods

1) calculation of the statistical factor

The association of fluoride was associated to the equilibria :

$$[ErL(H_2O)]^+ + F^- \leftrightarrow [ErLF] + H_2O \qquad K_{1\,Er} = \frac{[ErLF]\times[H_2O]}{[ErL(H_2O)]\times[F^-]} \quad (1)$$

$$[ErL(H_2O)]^+ + [ErLF]^+ \leftrightarrow [(ErL)_2F]^+ + H_2O \qquad K_{2\,Er} = \frac{[(ErL)_2F]\times[H_2O]}{[ErL(H_2O)]\ \times[ErLF]} \quad (2)$$

Following the work of Ercolani and coworkers,[1,2] the statistical factors for each equilibrium are:

$$K_1 = \frac{\sigma_{[ErL(H2O)]}\times\sigma_F}{\sigma_{[ErLF]}\times\sigma_{H2O}} \quad (3) \quad \text{and } K_2 = \frac{\sigma_{[ErL(H2O)]}\times\sigma_{[ErLF]}}{\sigma_{[(ErL)2F]}\times\sigma_{H2O}} \quad (4)$$

Assuming that the complexes undergo fast helical interconversion on the thermodynamic timescale of the titration experiments, the following symmetry numbers have to be taken into account :

$[ErL(H_2O)]^+$: C_{2v} point group, external symmetry number : 2; internal symmetry number : 2 (free rotation around the Er-O bond of the water molecule); total symmetry number : 4.

[(ErL)F]: C_{2v} point group, external symmetry number : 2; internal symmetry number : 1; total symmetry number : 2.

F^-: R_3 point group, external symmetry number : 1; internal symmetry number : 1; total symmetry number : 1.

$[(ErL)_2F]^+$: S_4 point group, external symmetry number : 2; internal symmetry number : 2; total symmetry number : 4.

H_2O: C_{2v} point group, external symmetry number : 2; internal symmetry number : 1; total symmetry number : 2.

Calculation of the statistical factors then give :

K_1 = (4×1)/(2×2) = 1 and K_2 = (4×2)/(4×2) = 1 and the ratio of the statistical factors is 1.

Supplementary References

(1) Ercolani, G.; Piguet, C.; Borkovec, M.; Hamacek, J. Symmetry numbers and statistical factors in self-assembly and multivalency *J. Phys. Chem. B*, **2007**, *111*, 12195-12203.

(2) Ercolani, G.; Schiaffino, L. Allosteric, chelate, and interannular cooperativity : A mise au point *Angew. Chem. Int. Ed.* **2011**, *50*, 1762-1768.
